# NH_4_^+^-Modulated Cathodic Interfacial Spatial Charge Redistribution for High-Performance Dual-Ion Capacitors

**DOI:** 10.1007/s40820-025-01660-0

**Published:** 2025-01-27

**Authors:** Yumin Chen, Ziyang Song, Yaokang Lv, Lihua Gan, Mingxian Liu

**Affiliations:** 1 Shanghai Key Lab of Chemical Assessment and Sustainability, School of Chemical Science and Engineering, Tongji University, Shanghai, 200092 People’s Republic of China; 2 College of Chemical Engineering, Zhejiang University of Technology, Hangzhou, 310014 People’s Republic of China

**Keywords:** NH_4_^+^-modulated cathodic interface, Spatial charge redistribution, Zn^2+^/NH_4_^+^ co-storage, Dual-ion capacitor

## Abstract

Compared with Zn^2+^, the current mainly reported charge carrier for zinc hybrid capacitors, small-hydrated-sized and light-weight NH_4_^+^ is expected as a better one to mediate cathodic interfacial electrochemical behaviors, yet has not been unraveled. Here we propose an NH_4_^+^-modulated cationic solvation strategy to optimize cathodic spatial charge distribution and achieve dynamic Zn^2+^/NH_4_^+^ co-storage for boosting Zinc hybrid capacitors. Owing to the hierarchical cationic solvated structure in hybrid Zn(CF_3_SO_3_)_2_–NH_4_CF_3_SO_3_ electrolyte, high-reactive Zn^2+^ and small-hydrate-sized NH_4_(H_2_O)_4_^+^ induce cathodic interfacial Helmholtz plane reconfiguration, thus effectively enhancing the spatial charge density to activate 20% capacity enhancement. Furthermore, cathodic interfacial adsorbed hydrated NH_4_^+^ ions afford high-kinetics and ultrastable C‧‧‧H (NH_4_^+^) charge storage process due to a much lower desolvation energy barrier compared with heavy and rigid Zn(H_2_O)_6_^2+^ (5.81 vs. 14.90 eV). Consequently, physical uptake and multielectron redox of Zn^2+^/NH_4_^+^ in carbon cathode enable the zinc capacitor to deliver high capacity (240 mAh g^−1^ at 0.5 A g^−1^), large-current tolerance (130 mAh g^−1^ at 50 A g^−1^) and ultralong lifespan (400,000 cycles). This study gives new insights into the design of cathode–electrolyte interfaces toward advanced zinc-based energy storage.

**Supplementary Information:**

The online version contains supplementary material available at 10.1007/s40820-025-01660-0.

## Highlights

- Hierarchical Zn^2+^/NH_4_^+^ solvation structure induces cathodic interfacial Helmholtz plane reconfiguration to enhance spatial charge density and capacity storage.
- Hydrated NH_4_^+^ ions afford high-kinetics and ultrastable C‧‧‧H charge storage due to a much lower desolvation energy barrier compared with large-sized Zn(H_2_O)_6_^2+^ (5.81 vs. 14.90 eV).
- Interfacial Zn^2+^/NH_4_^+^ co-storage endow the hybrid capacitor with high capacity (240 mAh g^−1^), large-current tolerance (50 A g^−1^) and ultralong lifespan (400,000 cycles).

**Supplementary Information:**

The online version contains supplementary material available at 10.1007/s40820-025-01660-0.

## Graphical abstract

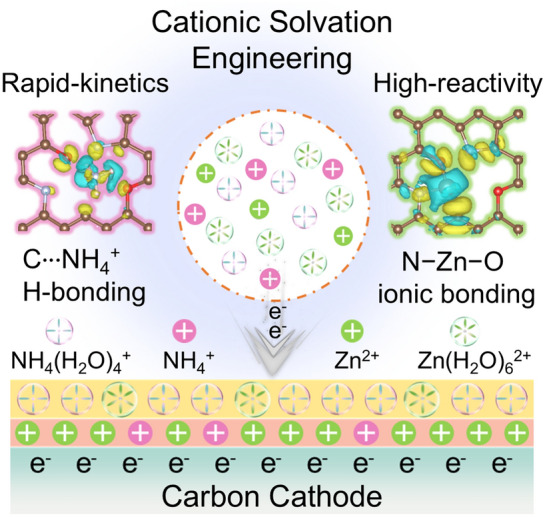

## Introduction

Aqueous zinc-ion hybrid capacitors (ZHCs) have recently emerged as highly competitive power-storage candidates due to their inherited dual superiorities from battery-type Zn anodes and supercapacitor-type carbon-based cathodes [1–5]. The reversible deposition/stripping behavior of Zn anode delivers high theoretical gravimetric capacity (820 mAh g^−1^) and suitable redox potential (− 0.76 V vs. the standard hydrogen electrode), providing sufficient charges for electrochemical energy storage [6–11]. Therefore, significant efforts have been made to develop high-performance cathode materials for propelling ZHCs, mainly focusing on customizing carbon nanostructures [12–17]. However, the developed carbon cathodes still suffer from an energy storage plafond at the electrode–electrolyte interfaces due to intrinsic inadequate zincophilic activity and unsustainable adsorption behavior, which hinder the performance improvement of ZHCs [18–20]. To cope with these dilemmas, the key breakthrough lies in designing highly electroactive and stable cathode–electrolyte interfaces to achieve more efficient charge storage.

Capacitive energy storage of carbon cathodes mainly relies on the electric double-layer (EDL) mechanism, which is largely determined by the specific surface area (SSA) and the distribution of charge carriers in electrolytes [21–24]. A large SSA can effectively expand electrode/electrolyte contact and carbon cathode capacity [25–29]. Nevertheless, the SSA of carbon cathodes is difficult to increase indefinitely, accompanied by negative effects such as inaccessible pores and poor pore-ion compatibility, leading to limited capacity enhancement [30, 31]. Charge carriers play an essential role in regulating the dynamic charge transfer and spatial storage at cathode interfaces, where high-density interfacial charge is beneficial for increasing carbon cathode capacity [12, 32, 33]. In this regard, the size and solvation structure of charge carriers considerably affect interfacial electrochemical behavior and EDL energy storage [34]. Thus, the spatial distribution of charge carriers at the cathode–electrolyte interface is particularly critical for efficient charge storage but has never been unraveled.

Metallic Zn^2+^ ions usually behave as highly active charge carriers for ZHCs, but their large hydrated structure and high desolvation energy led to a sluggish interfacial charge storage process, especially at high currents [35, 36]. In contrast, non-metallic NH_4_^+^ charge carriers show smaller hydrate size and lighter weight, giving enhanced dehydration and rapid reaction kinetics [37–40]. Moreover, NH_4_^+^ ions possess beneficial tetrahedral geometry and strong preferential orientation. Their interfacial non-metallic H-bonding interaction with cathodes is quite flexible compared with heavy and rigid Zn^2+^ ions, exhibiting a strong vitality to overcome the kinetics and stability hurdles. The respective structure/function originality of (non-)metallic charge carriers inspire us to consider whether high-active Zn^2+^ and high-kinetics NH_4_^+^ ions can serve as a powerful protocol for synergistically reconfiguring the cathodic-electrolyte interface spatial charge distribution and achieve efficient charge storage.

Herein, we propose a NH_4_^+^-modulated cationic solvation strategy to optimize spatial charge distribution and afford dynamic Zn^2+^/NH_4_^+^ co-storage for improving the rate and cycling metrics of ZHCs. Featured with hierarchical solvated cation structure in Zn(OTF)_2_–NH_4_OTF hybrid electrolyte (CF_3_SO_3_ = OTF), high-reactive Zn^2+^ and small-hydrate-sized NH_4_^+^ charge carriers can be redistributed to enable Helmholtz plane reconfiguration for effectively improving the spatial charge density and capacitive response (20% capacity enhancement). Moreover, NH_4_(H_2_O)_4_^+^ ions afford high-kinetics and ultrastable charge storage process due to a much lower desolvation energy obstacle compared with large and rigid Zn(H_2_O)_6_^2+^ ions (5.81 vs. 14.90 eV). As a consequence, physical uptake and multielectron redox of Zn^2+^/NH_4_^+^ in carbon cathode empower ZHCs with high-rate capacities and long-term cyclic stability. This work lays the foundation for designing cationic electrolytes for advanced ZHCs.

## Experimental Section

### Material Synthesis

#### Preparation of Aqueous Electrolytes

Different amounts of Zn(OTF)_2_ and NH_4_OTF salts were dissolved into deionized water, to obtain aqueous electrolytes of 2 M NH_4_OTF, 2 M Zn(OTF)_2_ and 1 M NH_4_OTF + 1 M Zn(OTF)_2_.

#### Synthesis of Zn-MOFs (MET-6)

In a typical synthesis, 3.0 g of ZnCl_2_ was dissolved in a mixed solvent comprising ethanol (30 mL), deionized water (50 mL), ammonium hydroxide (12 mL, 25%–28%) and N,N-dimethylformamide (DMF, 30 mL). Subsequently, 4 mL of 1H-1,2,3-triazole was gradually added to the solution under stirring at room temperature for 12 h. All these chemicals were purchased from Adamas-beta without any purification. Then the product obtained was filtered and washed thoroughly with ethanol and then dried at 80 °C for 12 h, resulting in the formation of a white powder (MET-6).

#### Synthesis of Porous Functional Carbon (PFC)

The synthesized MET-6 precursor was subjected to annealing at 800 °C for 2 h under a nitrogen atmosphere, with a heating rate of 2 °C min^–1^. The resulting carbonized material was treated with 4 M HCl for 4 h to remove the zinc components, followed by thorough washing with water and drying at 80 °C for 24 h. PFC@Zn was obtained in the same procedure without hydrochloric acid etching.

### Characterizations

The size distribution and ionic conductivity of electrolytes were characterized via dynamic light scattering instrument and zeta potential (Litesizer 500). Field-emission scanning electron microscopy (SEM, Hitachi S-4800) and transmission electron microscopy (TEM, JEM-2100) with an integrated X-ray energy-dispersive spectroscopy (EDS) system were utilized to examine the morphologies and elemental distributions. X-ray photoelectron spectroscopy (XPS, AXIS Ultra DLD) was employed to analyze the surface elemental composition and chemical states of the samples. Fourier-transform infrared (FT-IR) spectroscopy was conducted using a Thermo Nicolet NEXUS spectrometer. Nitrogen adsorption–desorption measurements were performed with a Micromeritics ASAP2020 analyzer to determine the Brunauer–Emmett–Teller (BET) surface area and pore size distribution.

*Ex* situ spectroscopic techniques, including XPS, SEM and FT-IR, were employed to investigate the surface chemistry of the cathode. Cathode samples were prepared by disassembling the batteries at specific voltages during the (de)charging process. The electrodes were thoroughly washed with distilled water to remove glass fibers and residual electrolytes. Subsequently, the cleaned electrodes were dried overnight in a vacuum oven at 60 °C to prevent oxidation of the samples.

### Electrochemical Measurements

The cathode was fabricated by mixing the PFC active material (70 wt%), acetylene black (20 wt%) and polytetrafluoroethylene binder (10 wt%) into a uniform slurry. The mixture was then coated onto a titanium foil and dried under vacuum at 110 °C for 12 h. The mass loading of the electroactive material was approximately 2.5 mg m^−2^. Zn(OTF)_2_–NH_4_OTF hybrid electrolytes, Zn metal anode (> 99.99%) and glass fiber separator were coupled into a 2032 coin-type cell. Polyacrylamide (PAM) gel is prepared according to the previously reported [21]. The prepared PAM dry hydrogel is immersed in Zn(OTF)_2_–NH_4_OTF hybrid electrolyte as the gel electrolyte. Flexible ZHCs (FZHCs) were assembled by sandwiching a PAM hydrogel electrolyte between a Zn foil and carbon cloth loaded with PFC cathode. Galvanostatic charge/discharge (GCD), rate performance and cycling stability tests were conducted using a Neware battery testing system (CT-4008Tn-5V10mA-164, Shenzhen, China). Cyclic voltammetry (CV) and electrochemical impedance spectroscopy (EIS) measurements were performed on a CHI660E electrochemical workstation, with an amplitude of 0.005 V and a frequency range of 10^–2^−10^6^ Hz. The specific capacity (*C*_m_, mAh g^−1^) was calculated from the GCD curves using the following equation:

1 $${{C}}_{\text{m}} = \frac{I \times \Delta}{m} $$

where *I* refer to the current density (A), Δ*t* represents the discharging time (s) and *m* is the mass of the loading cathode (g).

The energy density (*E*, Wh kg^–1^) and power density (*P*, W kg^–1^) are calculated according to the following equations:

2 $$ E  =  C_{{\text{m}}} \times \, \Delta V $$

3 $${{P}} = \frac{E}{1000 \times \Delta t} $$

where Δ*V* is the voltage window (V), Δ*t* represents the discharging time (h).

### Calculation Methods

#### Molecular Dynamics (MD) and Density Functional Theory (DFT) Simulations

The MD simulations were conducted using the Forcite module in Materials Studio 2020 [41, 42]. The COMPASS III force field was used along with optimized atom types and charges. The adsorption density isosurfaces were calculated using the Sorption module with the Metropolis method at ultrafine quality. The sorbate was exposed to pressures between 1 and 1000 kPa at 298 K. The Dreiding force field was employed, with charge calculations performed using the charge equilibration method and electrostatic interactions treated via Ewald summation. The calculations of the DFT were performed using the CASTEP. The generalized gradient approximation (GGA) Perdew–Burke–Ernzerhof (PBE) as the exchange correlation functional. The energy cutoff and SCF tolerance were 570 eV and 1 × 10^–6^ eV atom^–1^, respectively. The adsorption energy and desolvation energy model were calculated after the geometry optimization. The adsorption energy and desolvation energy Δ*E* were calculated according to the following equation:

4 $$ \Delta E = E_{{{\text{A}}/{\text{B}}}} - E_{{\text{B}}} {-}E_{{\text{A}}} $$

The electron localization function (ELF) was calculated by using Multiwfn 3.8 programs [43]. The molecular orbital levels (HOMO and LUMO) and charge population sums of metallic Zn^2+^ and non-metallic NH_4_^+^ were calculated at the B3LYP-D3/TZVP level to investigate the system's electronic properties. The independent gradient model (IGM) simulations were conducted via the Multiwfn program to investigate the type of interaction force when the value of sign(*λ*_2_)*ρ* approaches zero.

#### Activation Energy

The activation energy (*E*_a_, eV) of the charge transfer process was calculated from the Arrhenius equation:

5 $$ {1}/R_{ct} = A{\text{exp}}\left( { - E_{{\text{a}}} /RT} \right) $$

where *R*_ct_ is the charge transfer resistance (Ω), *A* is a constant in a stable experimental condition, *R* is the gas constant (8.314 J mol^−1^ K^−1^). Plot ln(*R*_ct_^−1^) versus 1000/*T* and fit it linearly to obtain *E*_a_:

6 $$ {\text{ln}}\left( {R_{{{\text{ct}}}}^{{ - {1}}} } \right) \, = - E_{{\text{a}}} /RT + k $$

where *k* is a constant.

**Fig. 1:**
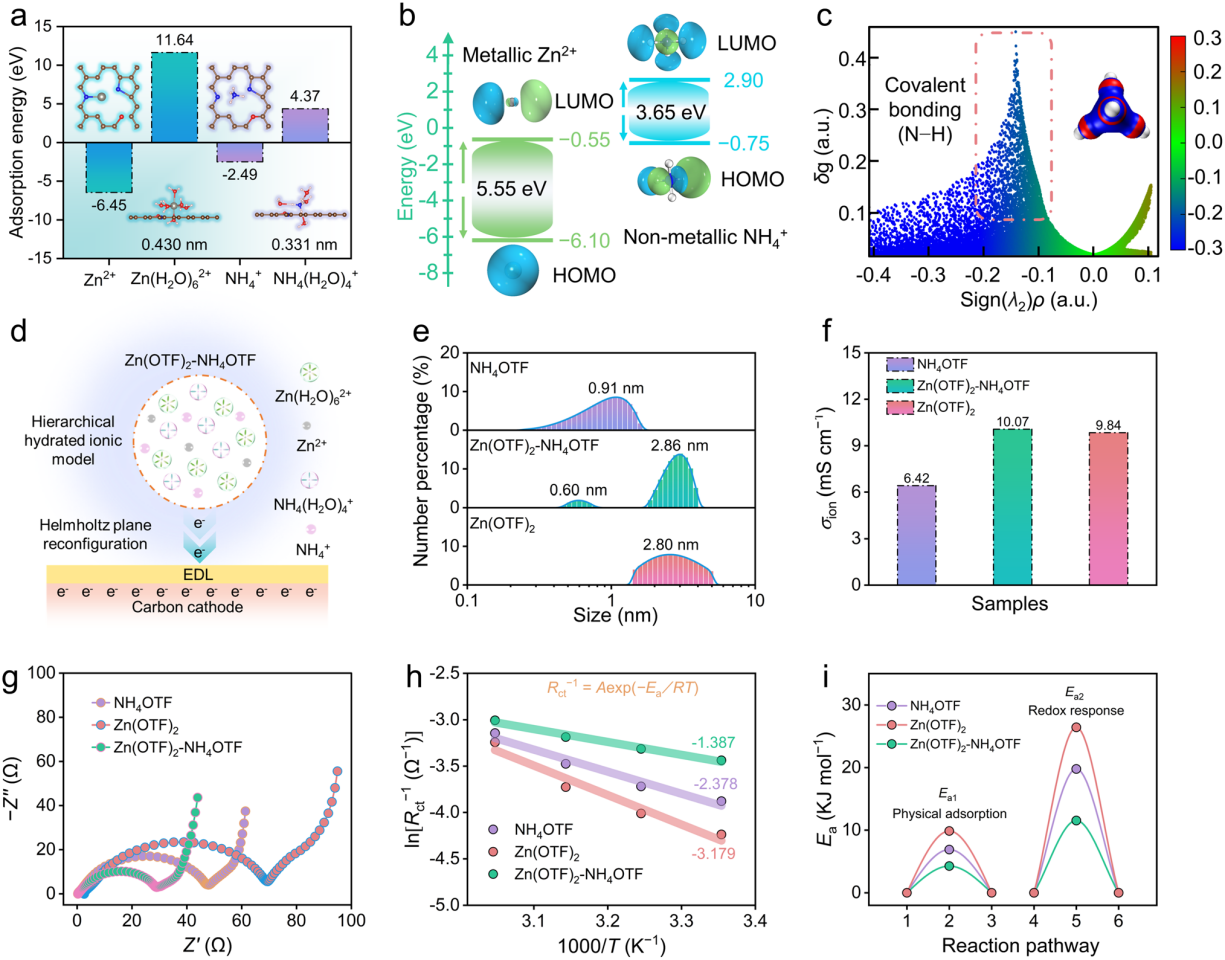
Property analysis of Zn^2+^ and NH_4_^+^ charge carriers. **a** Molecular adsorption model and relative energy. **b** Energy levels and frontier molecular orbitals. **c** Scatter plots of IGM. **d** Schematic interface adsorption model of Zn(OTF)_2_–NH_4_OTF hybrid electrolyte. **e** Size distributions of solvated cation aggregates. **f** Electrolyte conductivities. **g** EIS spectra. **h** Calculated *E*_a_ values and **i** reaction energy comparison

In order to calculate *E*_a_ values for the coordination processes with NH_4_^+^ and Zn^2+^, the electrochemical experiments of Zn||PFC capacitors were carried out in 2 M NH_4_OTF, 2 M Zn(OTF)_2_ and 1 M Zn(OTF)_2_ + 1 M NH_4_OTF hybrid electrolytes (Fig. 1h), respectively.

#### Charge Storage Kinetics

The sweep rate (*v*) and peak current (*i*) of ZHCs batteries were investigated based on the equation:

7 $$ i = av^{{\text{b}}} $$

they can be established by calculating the equation that follows between *i* and *V:*

8 $$ i = k_{{1}} v + k_{{2}} v^{{{1}/{2}}} $$

where *k*_1_ and *k*_2_ are constants,* k*_1_*v* represents the fast-capacitive process and *k*_2_*v*^1/2^ accounts for the diffusion-controlled process.

The combined series resistances (*R*_s_) can be extracted from the intersection of the curve and horizontal axis, comprising the electrolyte ionic resistance, electrode/electrolyte interface resistance and active material electronic resistance. The charge transfer resistance (*R*_ct_) refers to the radius of semicircles in the curves of Nyquist plots. The capacitance *C* (*ω*) changes along with the frequency which is defined as follow equations:

9 $$ C(\omega ) \, = C^{\prime}(\omega ) \, - jC^{\prime\prime}(\omega ) $$

10 $$ C^{\prime}\left( \omega \right) \, = \, {-}Z^{\prime\prime}\left( \omega \right)/\left( {\omega |Z\left( \omega \right)|^{{2}} } \right) $$

11 $$ C^{\prime\prime}\left( \omega \right) = Z^{\prime}\left( \omega \right)/\left( {\omega |Z\left( \omega \right)|^{{2}} } \right) $$

where *C*′(*ω*) is the real part of *C*(*ω*), the low frequency value of *C*′(*ω*) refers to the capacitance of the device tested in constant-current discharge process; *C*′(*ω*) represents the imaginary part associated with energy dissipation due to an irreversible process, resulting in hysteresis. *Zꞌ*(*ω*) and *Zꞌꞌ*(*ω*) are the real and imaginary parts of the complex impedance *Z*(*ω*), respectively. *ω* denotes the angular frequency, defined as *ω* = 2*πf*. The relaxation time constant (*τ*_0_) is calculated using the following equation:

12 $$ \tau_{0} = { 1}/f_{0} $$

*τ*_0_ is defined as the time constant at the frequency *f*_0_, where the imaginary part of the capacitance (*C*ꞌꞌ) reaches its maximum value.

## Results and Discussion

### Charge Carrier Properties

Charge carriers are one of the most important factors determining the electrochemical metrics of ZHCs [37, 44]. Among various metallic ions (Li^+^, Na^+^, Mg^2+^, Al^3+^, Ca^2+^ and Zn^2+^), Zn^2+^ charge carrier shows the highest reaction activity (− 6.45 eV, Fig. S1) with the simulated carbon framework, which does a favor to high-density spatial charge storage. However, compared to hydrated zinc ions, non-metallic NH_4_^+^ ions deliver a very small-hydrated radius (0.331 nm) and light molar mass. Moreover, small-sized NH_4_(H_2_O)_4_^+^ shows a lower repulsive force (4.37 eV) than that of larger-sized Zn(H_2_O)_6_^2+^ (11.64 eV, Figs. 1a and S2) in simulated carbon framework, benefiting rapid diffusion kinetics in aqueous electrolytes [45]. The favorable tetrahedral structure of NH_4_^+^ provides a lower energy gap (Δ*E*) of 3.65 eV than that of Zn^2+^ (5.55 eV, Fig. 1b), which is conducive to the realization of high electronic conductivity and rapid charge transfer. Compared with rigid spherical Zn^2+^ ions, flexible tetrahedral NH_4_^+^ ions offer quadruple N–H-bonds as demonstrated by the independent gradient model (IGM) as a function of the density, affording more adsorption sites for the formation of H-bonds with carbon cathodes (Fig. 1c) [46–48]. Nevertheless, bare NH_4_^+^ ion confers a considerable radius to trigger a relatively loose Helmholtz plane (Fig. S2), which is unable to fulfill high-energy density spatial charge storage. The specific and functional originality of charge carriers inspires us to use highly active Zn^2+^ and highly kinetic NH_4_^+^ ions as a powerful protocol to synergistically reconfigure the spatial charge distribution at the cathodic-electrolyte interface and achieve efficient charge storage.

Based on the ionic conductivity, electrochemical impedance and capacitance response of Zn(OTF)_2_–NH_4_OTF hybrid electrolytes with different proportions (Fig. S3), the optimized 1 M Zn(OTF)_2_ + 1 M NH_4_OTF electrolyte is designed to reconstruct the EDL at the cathode–electrolyte interface by combining high-reactive Zn^2+^ and rapid kinetics-hydrated NH_4_^+^ ions (Fig. 1d). Dynamic light scattering characterization (DLS) shows the size distribution of solvated cation aggregate in three different electrolytes (Fig. 1e) [49–52]. Zn(OTF)_2_–NH_4_OTF hybrid electrolyte exhibits a hierarchical hydrated cation structure with average sizes of 0.60 and 2.86 nm, which improve pore accessibility of carbon cathodes. In addition, Zn(OTF)_2_–NH_4_OTF hybrid electrolyte shows an excellent ionic conductivity (*σ*_ion_) of 10.07 mS cm^−1^ (Fig. 1f), superior to NH_4_OTF (6.42 mS cm^−1^) and Zn(OTF)_2_ (9.84 mS cm^−1^) electrolytes. Moreover, Zn(OTF)_2_–NH_4_OTF electrolyte has better hydrophilic properties with a contact angle of 46.8°, and this value is smaller than that of the NH_4_OTF electrolyte and Zn(OTF)_2_ electrolyte (Fig. S4). Fourier-transformed infrared (FT-IR) spectra show a redshift of O−H species due to the formation of H-bonds (N−H···O) between NH_4_^+^ ions and H_2_O molecules (Fig. S5). To evaluate the electrochemical properties of Zn(OTF)_2_–NH_4_OTF hybrid electrolyte, porous functional carbon (PFC) was prepared as the cathode material (Fig. S6), the characterization results demonstrate PFC carbon hierarchical structure and were doped with heteroatom. (Detail characterizations are shown in Figs. S6−S10.) The PFC cathode was couples with the Zn anode to construct ZHCs using different electrolytes. According to the electrochemical impedance spectroscopy (EIS) fitting results (Figs. 1g and S11a), the assembled zinc capacitor using hybrid Zn(OTF)_2_–NH_4_OTF electrolyte shows a low *R*_ct_ value (27.8 Ω), superior to NH_4_OTF electrolyte (48.3 Ω) and Zn(OTF)_2_ electrolyte (70.1 Ω). Moreover, the activation energy (*E*_a_) for the charge transfer procedure was estimated by EIS plots at different temperatures (Fig. S11b). *E*_a1_ corresponds to the physical adsorption of charged species, while *E*_a2_ is assigned to the redox response between heteroatomic motifs of carbon cathodes and electrolyte ions during electrochemistry [53]. Based on the Arrhenius equation (Eqs. S5 and S6) [54], Zn(OTF)_2_–NH_4_OTF electrolyte delivers very small *E*_a1_ and *E*_a2_ values of 4.3 and 11.5 kJ mol^−1^, respectively (Fig. 1h, i), which are lower than those of NH_4_OTF and Zn(OTF)_2_ (6.9−19.8 and 9.9−26.4 kJ mol^−1^). Moreover, the relaxation time constant (*τ*_0_, the time required for energy delivery) and the ion diffusion resistance (*σ*, fitting the linear relationship between the impedance real part (*Z'*) and the angular frequency (*ω*)) further confirm the superior charge transfer efficiency in the dual-cation electrolyte (Fig. S12 and Table S1). These results indicate high-kinetics charge storage activity of ZHCs with low reaction barriers, which benefit from high ionic conductivity and hierarchical solvated Zn^2+^/NH_4_^+^ structure of Zn(OTF)_2_–NH_4_OTF hybrid electrolyte.

### Cationic Solvation Structures

**Fig. 2:**
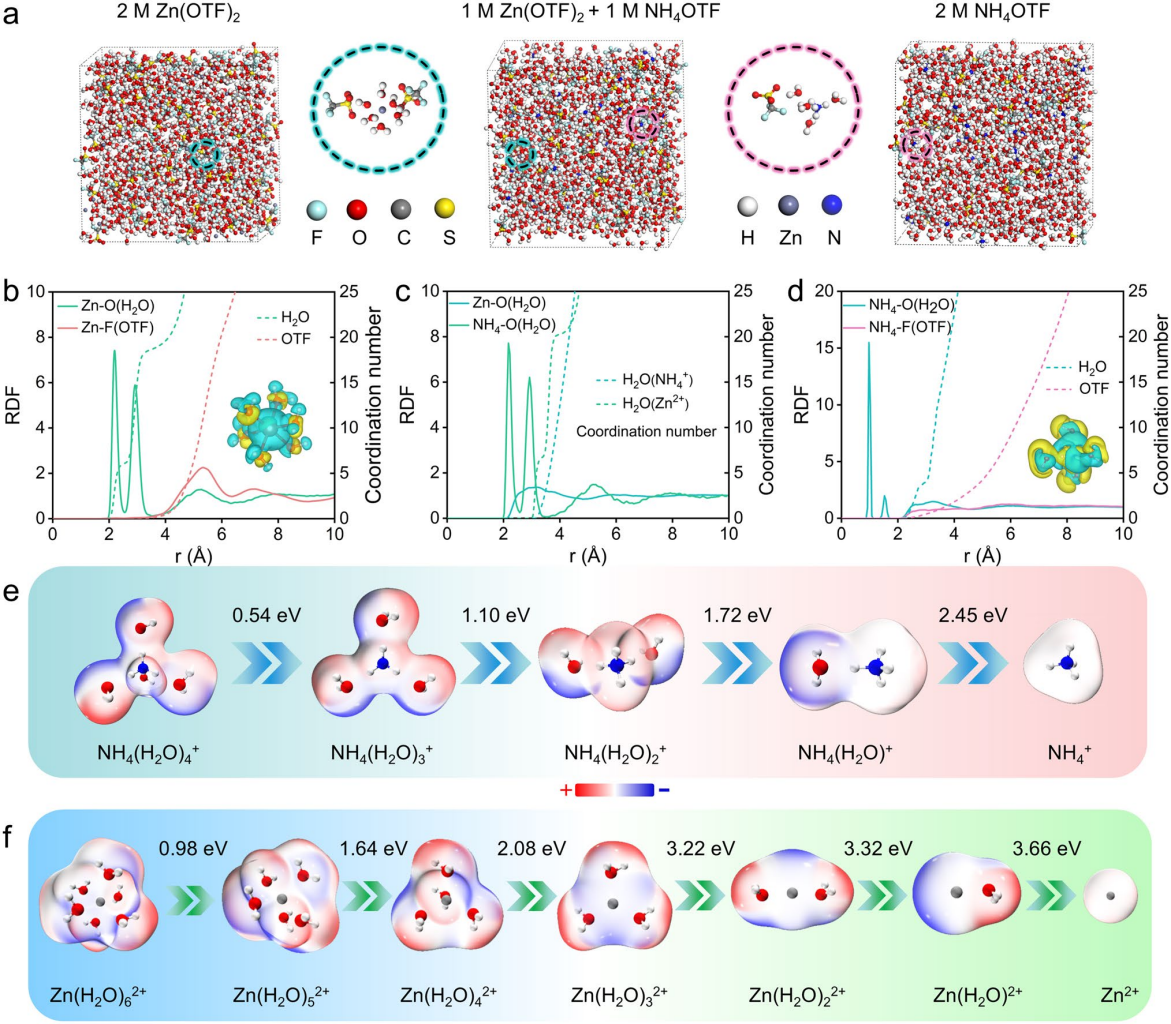
Solvation structures of three electrolytes. **a** 3D snapshots and corresponding enlarged images of solvation structures. RDF and CN plots of **b** 2 M Zn(OTF)_2_, **c** 1 M Zn(OTF)_2_–1 M NH_4_OTF and **d** 2 M NH_4_OTF electrolytes. Optimized MEP and desolvation energy barriers of **e** NH_4_^+^ and **f** Zn^2+^ coordination structures

To investigate the physicochemical properties of hybrid Zn(OTF)_2_–NH_4_OTF electrolyte, molecular dynamics (MD) simulations and density functional theory (DFT) calculations [55–59] were performed to get insight into the solvation structures of three different electrolytes. The 3D snapshots and corresponding enlarged images of solvation structures of three electrolytes are shown in Fig. 2a. Moreover, the radial distribution functions (RDF, solid lines) and coordination numbers (CN, dashed lines) for three electrolytes were calculated from the statistical averaging of the full-time snapshot data (Fig. 2b−d). In the first solvation sheath, Zn^2+^ ion can coordinate with six H_2_O molecules, together with obvious charge transfer in 2 M Zn(OTF)_2_ electrolyte and two OTF^−^ anions adsorbed outside the first solvation sheath (Fig. 2b). NH_4_^+^ couples with four H_2_O molecules in 2 M NH_4_OTF electrolyte via H-bonding interactions (Fig. 2d). In contrast, due to the competitive solvating effect of Zn^2+^ and NH_4_^+^ in Zn(OTF)_2_–NH_4_OTF hybrid electrolyte (Fig. 2c), small-hydrated-sized NH_4_^+^ ion is more likely to activate strong charge transfer compared with Zn^2+^ ion. Molecular electrostatic potential (MEP) distribution and desolvation energy of optimized solvated cation structures are shown in Fig. 2e, f. At identical applied external potential, NH_4_(H_2_O)_4_^+^ generally shows lower desolvation energy barriers in comparison with Zn(H_2_O)_6_^2+^ (Figs. S13−S16). It suggests that NH_4_(H_2_O)_4_^+^ is more likely to lose H_2_O molecule than Zn(H_2_O)_6_^2+^. As a result, desolvated small-sized Zn^2+^ ions with high reaction activity can be easily adsorbed inside PFC cathode, which helps to improve spatial charge storage density, while NH_4_^+^ ion with low desolvation energy barrier ensures fast reaction kinetics and high-power delivery.

### Cathode–Electrolyte Interfaces

**Fig. 3:**
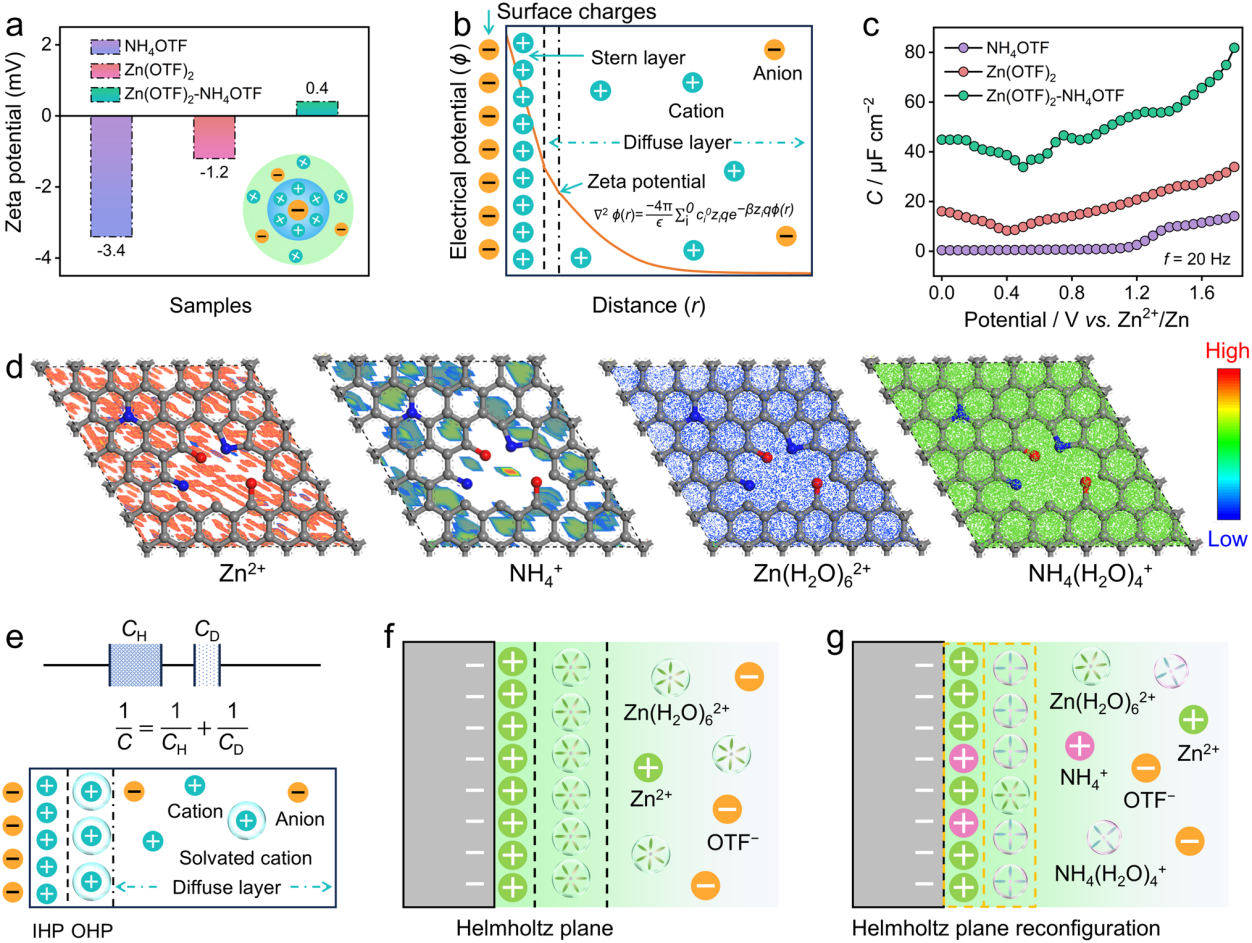
Investigation of PFC cathode–electrolyte interfaces. **a** Zeta potentials. **b** The relationship between zeta potential and EDL model. **c** Area-normalized differential capacitance potential curves. **d** Adsorption density isosurfaces of various charge carriers in PFC skeleton. **e** Formula of EDL capacitance and relative model. Spatial charge distributions of **f** Zn(OTF)_2_ and **g** Zn(OTF)_2_–NH_4_OTF electrolyte at the cathodic interfaces

The changes of zeta potential reflect the variation in spatial charge distribution and interfacial charge properties in different electrolytes, establishing the direct relationship between the adsorbed interfacial configurations and solvated structures [60, 61]. The zeta potentials show the disturbance of the potential field in three electrolytes (Fig. 3a), along with the increase of potential values from − 3.4 mV (NH_4_OTF) and − 1.2 mV (Zn(OTF)_2_) to 0.4 mV (Zn(OTF)_2_–NH_4_OTF). A positive potential shift indicates the enhanced cation adsorption ability with Helmholtz plane reconfiguration (Fig. 3b). Moreover, the differential capacitance (*C*) tests at varying applied potentials [23, 62] were further performed to study cationic adsorption behavior of different electrolytes. Among the three electrolytes, Zn(OTF)_2_–NH_4_OTF hybrid electrolyte exhibits the highest capacitance response (Fig. 3c), which can be attributed to the increased spatial charge density in the cathode–electrolyte interface. These results facilitate the establishment of the correlation between the size of solvated cations and the capacitive charge storage response.

Furthermore, we simulated the adsorption sites and densities of cathode–electrolyte interfaces (Figs. 3d and S17) [63, 64]. The surface of the PFC cathode exhibits a higher adsorption concentration of Zn^2+^ ions than that of NH_4_^+^ ions (Fig. S18). Intriguingly, the adsorption concentration of NH_4_(H_2_O)_4_^+^ is higher than that of Zn(H_2_O)_6_^2+^, which is consistent with the calculated adsorption energy (Fig. 1a). Based on these findings, the incorporation of NH_4_^+^ into Zn(OTF)_2_–NH_4_OTF hybrid electrolyte facilitates Helmholtz plane reconfiguration of PFC cathode, and the unique EDL adsorption-induced charge flow enables high availability of electrophilic regions and rapid ion migration to boost electrochemical energy storage. The interfacial adsorption model in Zn(OTF)_2_–NH_4_OTF electrolyte was elucidated based on the well-known Gouy–Chapman–Stern (GCS) model [22]. The EDL consists of a Helmholtz layer (compact) and a diffusion layer (loose) (Fig. 3e), where the capacitance (*C*_EDL_) is determined by the equation of 1/*C*_EDL_ = 1/*C*_H_ + 1/*C*_D_, where *C*_H_ and *C*_D_ represent the capacitance of the Helmholtz region and diffusion layer [65], respectively. As well-known, *C*_H_ contributes to most of the dielectric constant and *C*_H_ is much higher than *C*_D_ at the cathode surface. Hence *C*_EDL_ can be approximated as 1/*C*_EDL_ = 1/*C*_IH_ + 1/*C*_OH_, where* C*_IH_ and *C*_OH_ represent the inner Helmholtz plane (IHP) and outer Helmholtz plane (OHP) [66, 67], respectively. Thus, regulating the structures of IHP and OHP is an efficient route to improve the capacity of ZHCs. In Zn(OTF)_2_ electrolyte and their derived cathodic interfacial model (Fig. 3f), Zn^2+^ ions tend to be captured in the IHP, whereas large-sized Zn(H_2_O)_6_^2+^ ions lead to a loose OHP interface. Similarly, NH_4_^+^ ions of NH_4_OTF electrolyte in the IHP are responsible for an inadequate capacitive response (Fig. S19). Thus, the EDL structure is reconstructed in Zn(OTF)_2_–NH_4_OTF electrolyte (Fig. 3g), of which high-reactivity Zn^2+^ ions occupy in the IHP and small-size NH_4_(H_2_O)_4_^+^) ions tightly adsorb in the OHP, thus stimulate cathodic interfacial spatial charge redistribution and effectively improving charge storage density to increase capacity storage.

### Electrochemical Performances

**Fig. 4:**
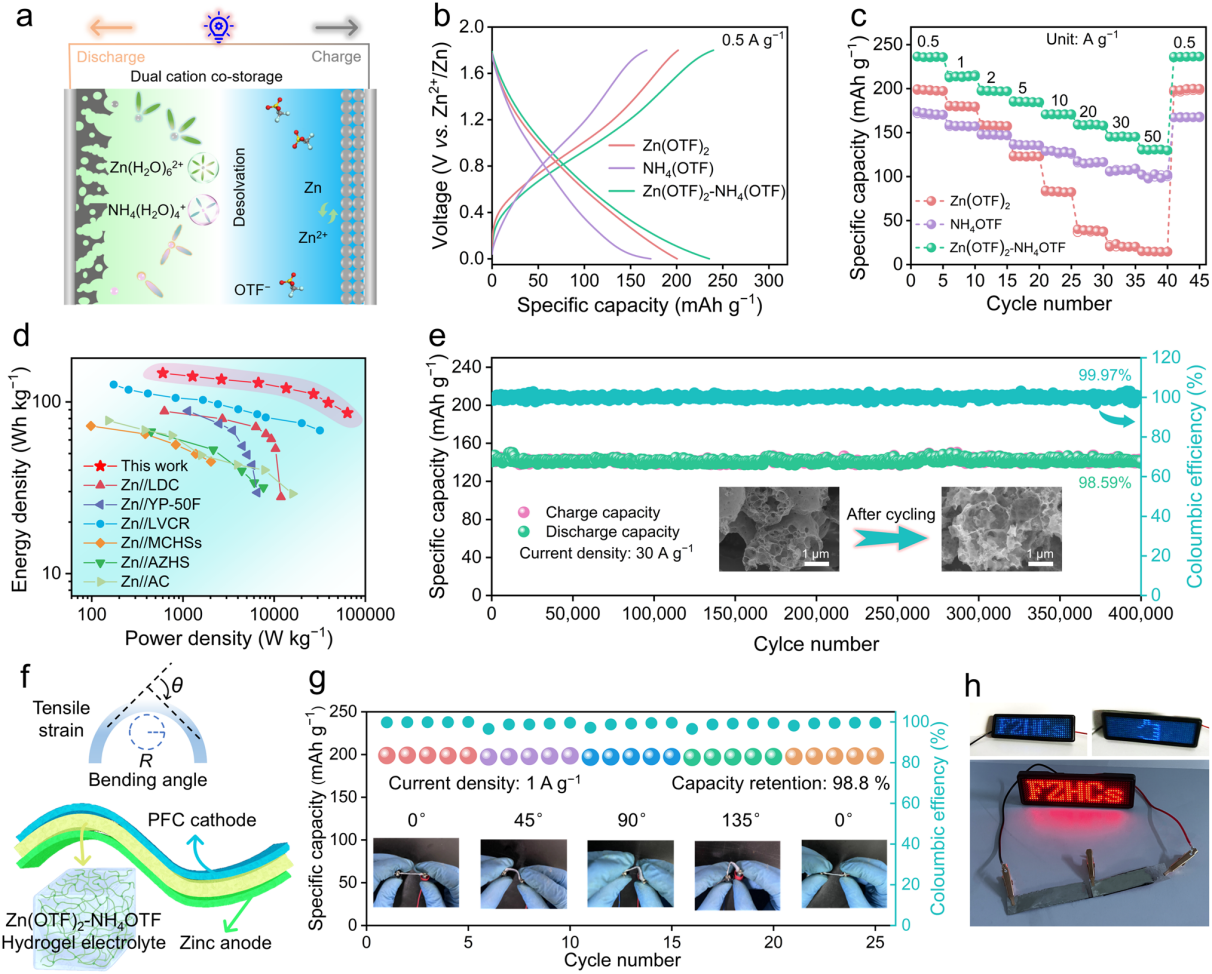
Electrochemical evaluation of Zn||PFC capacitors. **a** Schematic of charge storage mechanism. **b** GCD profiles. **c** Rate capabilities. **d** Ragone plots compared with reported Zn hybrid capacitors. **e** Cycling performance. **f** Schematic diagram of flexible Zn||PFC devices. **g** Flexible bending performance of FZHCs. **h** Optical image of an LED display panel powered by FZHCs

Schematic configuration of Zn||PFC capacitor is shown in Fig. 4a, which involves Zn metal anode, PFC cathode and aqueous electrolytes. Cyclic voltammetry (CV) curves of Zn||PFC capacitor using Zn(OTF)_2_–NH_4_OTF electrolyte exhibit the largest integral area (Fig. S20a), which can be attributed to the effectively compressed EDL thickness. A high discharge capacity of 240 mAh g^−1^ at 0.5 A g^−1^ is achieved for Zn||PFC capacitor in Zn(OTF)_2_–NH_4_OTF electrolyte (Figs. 4b and S20b−d), surpassing Zn(OTF)_2_ (198 mAh g^−1^) and NH_4_OTF (172 mAh g^−1^) electrolytes. Even at 50 A g^−1^, PFC cathode in Zn(OTF)_2_–NH_4_OTF electrolyte still holds a remarkable capacity of 130 mAh g^−1^ (15 mAh g^−1^ in Zn(OTF)_2_; 98 mAh g^−1^ in NH_4_OTF), unraveling superior fast reaction kinetics and rate capabilities (Fig. 4c). Compared with large-sized Zn^2+^ ions, small-hydrated-sized and light-weight NH_4_^+^ as a better one can enable high-kinetics cathodic interfacial reactions to deliver superior rate capacities at elevated current densities. The large capacity endows the assembled Zn||PFC capacitor in Zn(OTF)_2_–NH_4_OTF electrolyte with an impressive energy density of 147 Wh kg^−1^ (Fig. 4d, based on the mass loading of PFC in the cathode, 2.5 mg cm^−2^), which is higher than those of Zn(OTF)_2_ electrolyte (136 Wh kg^−1^) and NH_4_OTF electrolyte (120 Wh kg^−1^). In addition, the device delivers an outstanding power density of 61.1 kW kg^−1^. More encouragingly, Zn||PFC capacitor using Zn(OTF)_2_–NH_4_OTF electrolyte presents a splendid capacity retention of 98.59% over 400,000 cycles at 30 A g^−1^ (Figs. 4e and S21). SEM images and XRD analysis of PFC cathode after long-term cycling demonstrate its excellent morphological and structural stability (Figs. 4e and S20f). The stable PFC cathode and Zn anode synergistically propel the long-lasting operation of Zn hybrid capacitors (Fig. S22). Unique Helmholtz reconfiguration plane between PFC cathode and Zn(OTF)_2_–NH_4_OTF electrolyte interface ensures high-kinetics ion diffusion to unlock superior capacitive response, affording all-round boosted electrochemical metrics for Zn||PFC capacitor (Table S2).

The reaction kinetics of Zn||PFC capacitor in Zn(OTF)_2_–NH_4_OTF electrolyte was investigated based on Dunn's approach [13, 68, 69]. Quasi-rectangular CV curves show a pair of redox peaks (marked as P_O_ and P_R_, Fig. S23a), revealing double-layer capacity and pseudocapacity contribution. The relationship between current (*i*) and scan rate(*v*) can be expressed as *i* = *kv*^*b*^, where *k* is constant. Plotting log*i* against log*v* gives *b* values of 0.78 for P_O_ and 0.83 for P_R_ (Fig. S23b−d), indicating diffusion-controlled and capacitive charge storage behaviors. The capacitive contribution of Zn||PFC capacitor in Zn(OTF)_2_–NH_4_OTF electrolyte dominates the diffusion-controlled contribution, superior to Zn(OTF)_2_ and NH_4_OTF electrolytes (Figs. S24 and S25). The reconfigured Helmholtz plane in Zn(OTF)_2_–NH_4_OTF electrolyte accelerates ion migration with low energy barriers and affords robust charge storage kinetics.

Quasi-solid flexible Zn||PFC capacitor (FZHCs) using Zn(OTF)_2_–NH_4_OTF hydrogel electrolyte was assembled (Figs. 4f and S26), to demonstrate the application potential in flexible energy storage fields [70]. The assembled capacitor shows excellent flexibility and tensile strain ability with stable electrochemical capacities at bending angles from 0° to 135° (Fig. 4g). When the bending angle returns to the initial value, its electrochemical performance still maintains 98.80% of the initial capacity. Furthermore, the connection of two flexible Zn||PFC devices in series can power LED display panel (Fig. 4h), expanding its potential for flexible wearable applications.

### Charge Storage Mechanism

**Fig. 5:**
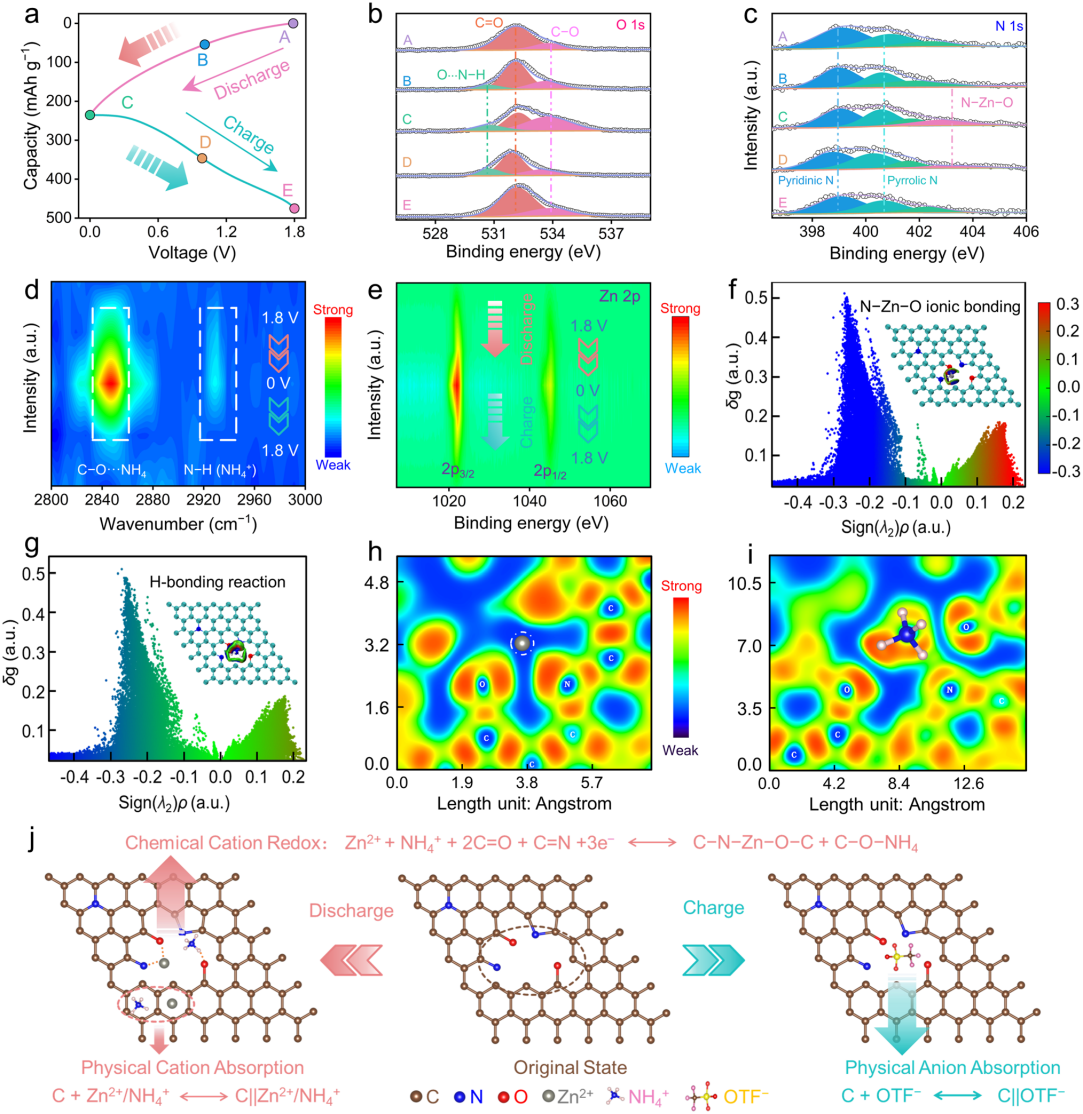
Charge storage behavior of Zn||PFC capacitor in Zn(OTF)_2_–NH_4_OTF electrolyte. **a** A GCD profile at 0.5 A g^−1^. Ex situ XPS spectra of **b** O 1*s* and **c** N 1*s*. **d** Ex situ FT‐IR spectra. **e** Zn 2*p* XPS spectra. Theoretical simulations of Zn^2+^ and NH_4_^+^ charge carriers stored in PFC cathode. Plots of IGMH versus sign(*λ*_2_)*ρ* and corresponding gradient isosurfaces of **f** Zn^2+^ and **g** NH_4_^+^ ions. ELF maps of **h** Zn^2+^ and **i** NH_4_^+^ ions in optimized carbon skeleton. **j** Charge storage mechanism of PFC cathode in Zn(OTF)_2_–NH_4_OTF electrolyte

To unveil the charge storage mechanism of PFC cathode in Zn(OTF)_2_–NH_4_OTF electrolyte, five marked (dis)charge states of GCD profile were selected for spectroscopic characterizations to monitor its structural variation (Fig. 5a). XPS and FT-IR spectra characterization were carried out to get insights into the reaction process. At initial state A, two deconvoluted peaks of O 1*s* spectra at 532.1 and 533.9 eV correspond to C=O and C−O (Fig. 5b). The C=O motif undergoes a continuous decay during discharging (state A → B → C) and strengthens in subsequent charging (state C → D → E), accompanied by reversible opposite changes of C−O motif. This result elucidates the strong activity of C=O motif in PFC cathode. Of note, a generated curve-fitted signal at 530.7 eV during discharging unravels the H-bonding reactions (O···H−N) [71]. Regarding the high-resolution N 1*s* spectra of PFC cathode, the deconvoluted spectra entail three signals at the original state A (Fig. 5c), attributing to pyridinic N (398.5 eV), pyrrolic N (400.2 eV) and quaternary N (402.5 eV) [72]. A new peak at 403.1 eV is detected during discharging, which assigns to O−Zn−N bond [73], indicating that pyridine/C=O groups can stimulate the uptake of Zn^2+^ ions on PFC cathode.

Based on ex situ FT-IR spectra, a new-emerged signal (2846 cm^−1^) can be observed (Fig. 5d), which gradually intensifies during discharging and weakens during subsequent charging. Such a variation corresponds to the H-bond stretching mode (O···H−N) between C=O motifs and NH_4_^+^ ions. The N−H peak of NH_4_^+^ at 2929 cm^−1^ further validates the formation/disappearance of the H-bonding interaction between NH_4_^+^ and C=O. Furthermore, Zn 2*p* signal gradually strengthens during discharging due to the enhanced Zn^2+^ adsorption on PFC cathode surface and almost disappears after recharging by releasing Zn^2+^ ions (Fig. 5e). On the contrary, S 2*p* signal decreases during discharging and returns to initial level after charging, suggesting OTF^−^ uptake/removal on PFC cathode (Fig. S27). Besides, the H^+^ uptake capacity (23.6 mAh g^−1^, Fig. S28) of Zn||PFC capacitor in HOTF electrolyte (pH = 3.31) is negligible. Moreover, SEM images of PFC cathode and zinc anode show excellent structure stability during cycling (Figs. S29−S31), ensuring highly reversible and stable energy storage.

DFT calculations were performed to get insight into the binding properties of PFC cathode upon Zn^2+^ and NH_4_^+^ uptake/removal. Reduced density gradient (IGMH) plots of Zn^2+^ and NH_4_^+^ stored in optimized PFC skeleton detect strong spikes (Fig. 5f, g), suggesting the interaction between Zn^2+^/NH_4_^+^ and PFC. At the same interaction region, Zn^2+^ ions show strong coupling ability with PFC cathode. Electron localization function (ELF) maps (Fig. 5h, i) and differential electron density isosurfaces (Fig. S32) reveal the bonding nature between carbonyl/pyridine motifs and Zn^2+^/NH_4_^+^ ions. Obviously, electrons are localized around carbonyl/pyridine motifs and almost absent around Zn atom, indicating their strong bonding character. The differential electron density isosurfaces confirm the charge transfer trend from adsorbed Zn^2+^/NH_4_^+^ to electroactive carbonyl/pyridine motifs. Bader charge analysis unravels more charge shifts of 0.62 e for Zn^2+^ (0.27 e for NH_4_^+^), indicating that Zn^2+^ ions are more likely to form favorable N−Zn−O bonds and NH_4_^+^ ions generate high-kinetics H-bonding reaction.

Overall, the Zn^2+^/NH_4_^+^ dual-ion co-storage mechanism is proposed for PFC cathode in Zn(OTF)_2_–NH_4_OTF electrolyte (Fig. 5j), which entails two types of electrochemical energy storage mode during discharging: (1) physical uptake of Zn^2+^/NH_4_^+^ on PFC cathode surface to yield EDL capacity; (2) multielectron redox of Zn^2+^/NH_4_^+^ with carbonyl/pyridine motifs to form O−Zn−N bonds and O···H−N H-bonds to deliver pseudocapacity. The subsequent charging course involves the physical adsorption of OTF^−^ at PFC cathode. Thus, the proposed cationic solvation strategy reconfigurate the interfacial Helmholtz planes for optimizing spatial charge distribution and achieves Zn^2+^/NH_4_^+^ co-storage for achieving high-performance zinc capacitors.

## Conclusions

In conclusion, a NH_4_^+^-regulated cationic solvation strategy is proposed to mediate dynamic charge transfer and functionalized redox response at the carbon cathode interface, which affords efficient Zn^2+^/NH_4_^+^ co-storage for enhancing both rate capabilities and cycling stability of ZHCs. The hierarchical cationic solvated structure in Zn(OTF)_2_–NH_4_OTF hybrid electrolyte facilitates interfacial charge carrier distribution and Helmholtz plane reconfiguration to improve space charge density and capacitance response. Moreover, the synergistic interfacial adsorption behavior of Zn^2+^/NH_4_^+^ ions is revealed, where Zn^2+^ ions and carbonyl/pyridine motifs are more prone to form favorable N−Zn−O bonds, while NH₄⁺ ions afford rapid kinetics via H-bonding reaction. As a consequence, the assembled zinc hybrid capacitor delivers high capacity, high-rate performance and excellent cyclic life. This work provides new insights into the rational engineering of cathode–electrolyte interfaces for advanced Zn-based energy storage.

## Supplementary Information

Supplementary file 1 (DOCX 6818KB)
