# Correction: NH_4_^+^-Modulated Cathodic Interfacial Spatial Charge Redistribution for High-Performance Dual-Ion Capacitors

**DOI:** 10.1007/s40820-025-01722-3

**Published:** 2025-04-09

**Authors:** Yumin Chen, Ziyang Song, Yaokang Lv, Lihua Gan, Mingxian Liu

**Affiliations:** 1 Shanghai Key Lab of Chemical Assessment and Sustainability, School of Chemical Science and Engineering, Tongji University, Shanghai, 200092 People’s Republic of China; 2 College of Chemical Engineering, Zhejiang University of Technology, Hangzhou, 310014 People’s Republic of China

**Correction to: Nano-Micro Letters (2025) 17:117** 10.1007/s40820-025-01660-0

Following publication of the original article [1], the authors reported that the supplementary file needed to be updated because they mistakenly used the incorrect version.

The original article [1] has been corrected.
