# Supplementary material for: NH4+-Modulated Cathodic Interfacial Spatial Charge Redistribution for High-Performance Dual-Ion Capacitors
Source: Nanomicro Lett. 2025 Jan 27;17:117. doi: 10.1007/s40820-025-01660-0 (PMC11772636; doi:10.1007/s40820-025-01660-0)
Supplement: Supplementary file 1 — (DOCX 6818KB) [file 40820_2025_1660_MOESM1_ESM.docx]

Supporting Information for

**NH_4_^+^-Modulated Cathodic Interfacial Spatial Charge Redistribution for High-Performance Dual-Ion Capacitors**

Yumin Chen^1^, Ziyang Song^1,^ *, Yaokang Lv^2^, Lihua Gan^1,^ *, Mingxian Liu^1,^ *

^1^ Shanghai Key Lab of Chemical Assessment and Sustainability, School of Chemical Science and Engineering, Tongji University, Shanghai 200092, P. R. China

^2^ College of Chemical Engineering, Zhejiang University of Technology, Hangzhou 310014, P. R. China

*Corresponding authors. E-mail: [21310240@tongji.edu.cn](mailto:21310240@tongji.edu.cn) (Ziyang Song); [ganlh@tongji.edu.cn](mailto:ganlh@tongji.edu.cn) (Lihua Gan); [liumx@tongji.edu.cn](mailto:liumx@tongji.edu.cn) (Mingxian Liu)

**Supplementary Figures**

**
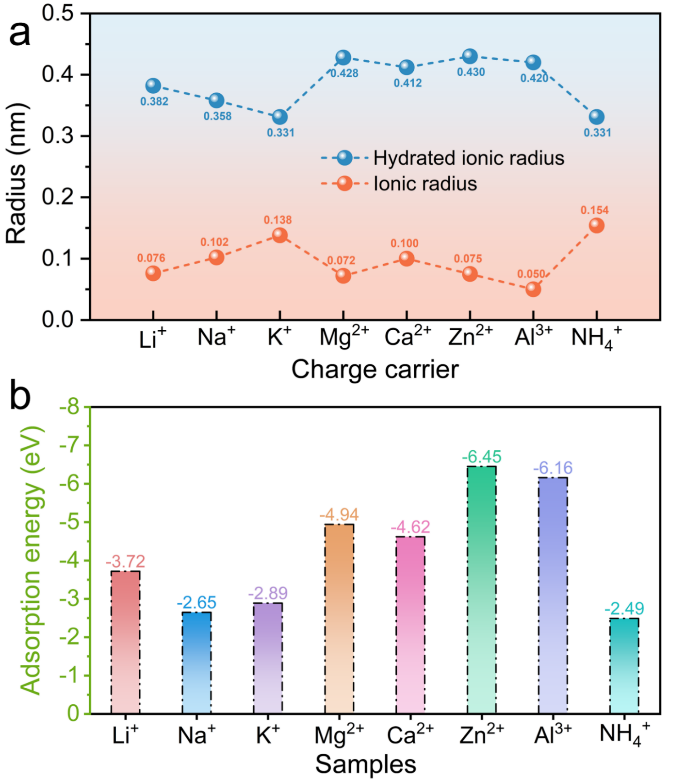
**

**
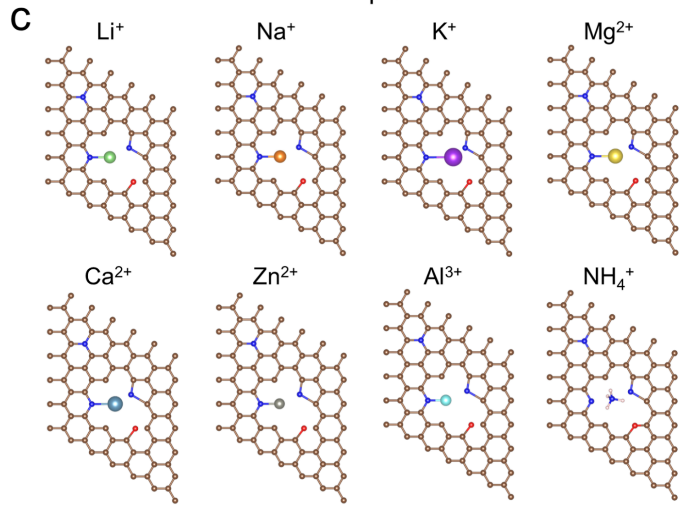
**

**Fig. S1** **a** Ionic radius and hydrated ionic radius of various charge carriers. **b** Reaction activity of various charge carriers. **c** Optimized structure model on a simulated carbon framework.

**
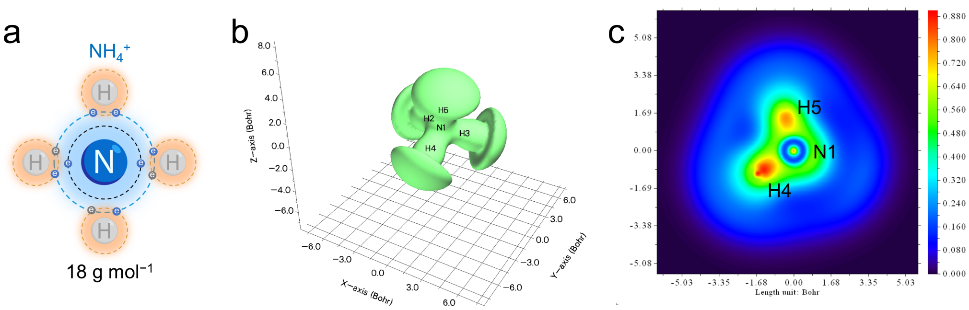
**

**Fig. S2 a,b** Molecular structure model of NH_4_^+^. **c** Localized orbital locator map of NH_4_^+^ in YZ plane.


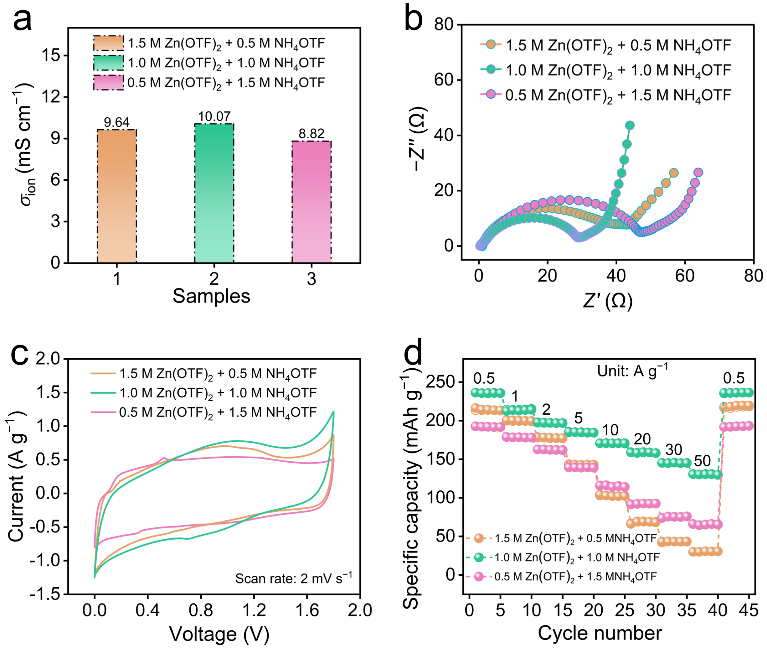


**Fig. S3** Electrochemical properties of Zn(OTF)_2_-NH_4_OTF hybrid electrolyte with different molar ratios: **a** electrolyte conductivities, **b** EIS spectra, **c** CV curves in different electrolytes and **d** rate performance.

**Notes to Fig. S3**: Zn(OTF)_2_-NH_4_OTF hybrid electrolytes with different proportions were modulated to explore the effect of NH_4_OTF concentration on electrochemical performances of Zn capacitors (Fig. S3). Among three various electrolytes, 1 M Zn(OTF)_2_ + 1 M NH_4_OTF electrolyte show the highest ionic conductivity (Fig. S3a) and lowest electrochemical impedance (Fig. S3b), thus delivering superior capacitive charge storage behaviors (Fig. S3c) and discharge capacities at different current densities (Fig. S3d). These results underline the critical role of NH_4_OTF in optimizing electrolyte properties and electrochemical metrics. The OTF-based-electrolyte was chosen owing to its following merits [S1-2]: i) OTF-based electrolytes exhibit excellent ionic conductivity due to the high dissociation ability of the trifluoromethanesulfonate (OTF^−^) anion, which facilitates efficient ion transport; ii) large-sized OTF^−^ anion has strong electrostatic interactions with Zn^2+^ ions, which helps to stabilize Zn deposition process, reduce dendrite formation and improve cycling stability; iii) OTF-based electrolytes have wide electrochemical stability windows. Considering the above factors, we selected Zn(OTF)_2_-NH_4_OTF as a promising electrolyte for propelling Zn hybrid capacitors.


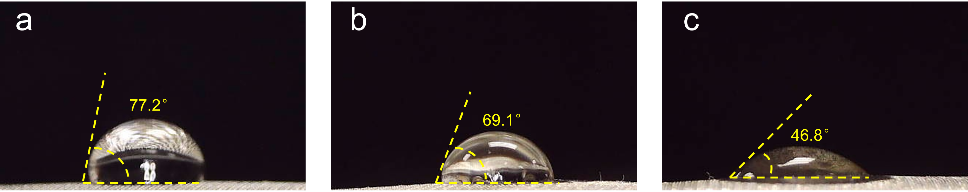


**Fig. S4** Contact angle measurement results of three electrolytes. **a** 2 M NH_4_OTF. **b** 2 M Zn(OTF)_2_. **c** 1 M Zn(OTF)_2_ + 1 M NH_4_OTF.


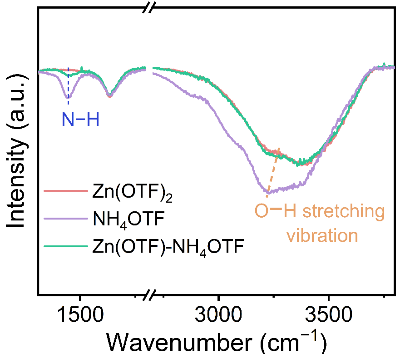


**Fig. S5** FT-IR spectra of three electrolytes.

**
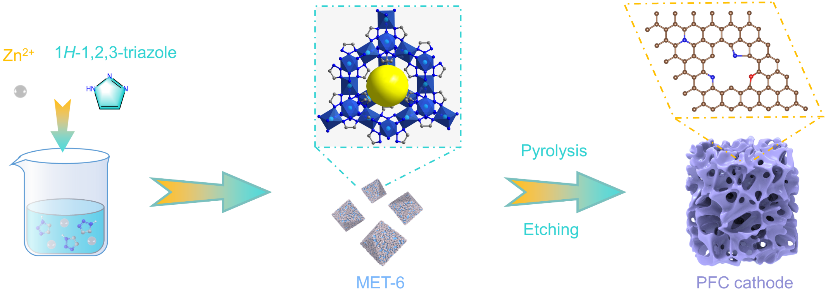
**

**Fig. S6** Schematic illustration of synthesis of PFC.

**
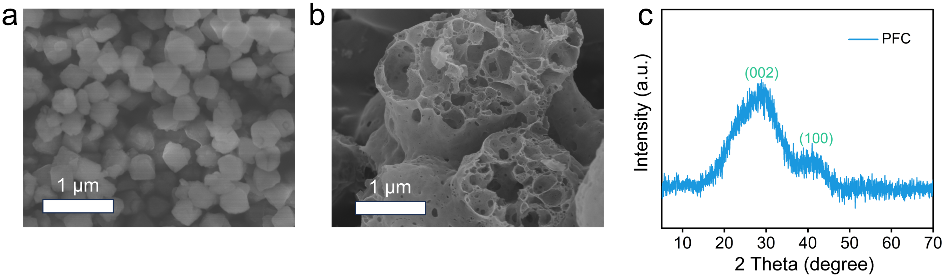
**

**Fig. S7** SEM images of **a** MET-6 and **b** PFC, and **c** XRD pattern of PFC.

**Notes to Fig. S7**: XRD pattern of PFC shows obvious peaks at 27.8° and 42.8° (Fig. S7c), which can be assigned to (002) and (100) diffraction planes.


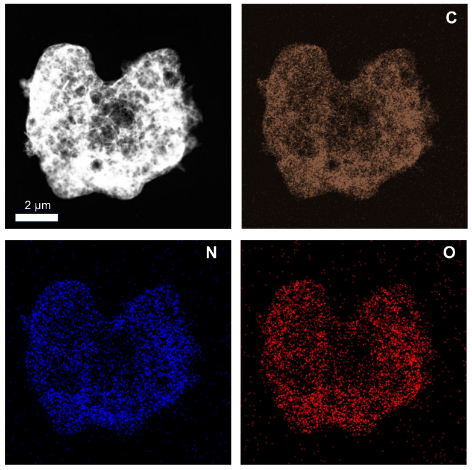


**Fig. S8** TEM-EDS maps of PFC cathode.

**
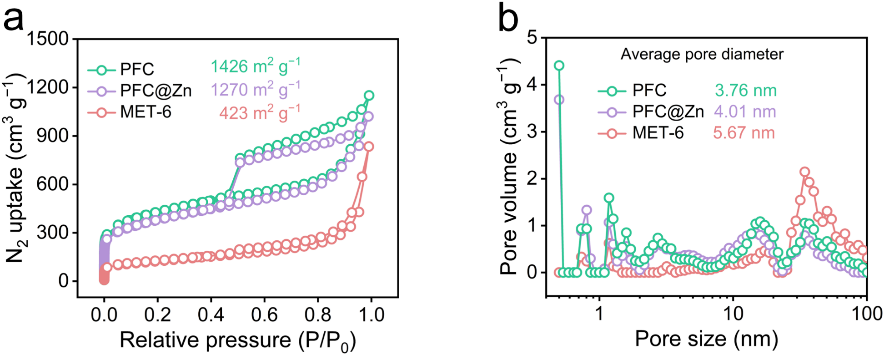
**

**Fig. S9 a** Nitrogen sorption isotherms and **b** corresponding pore size distribution curves.


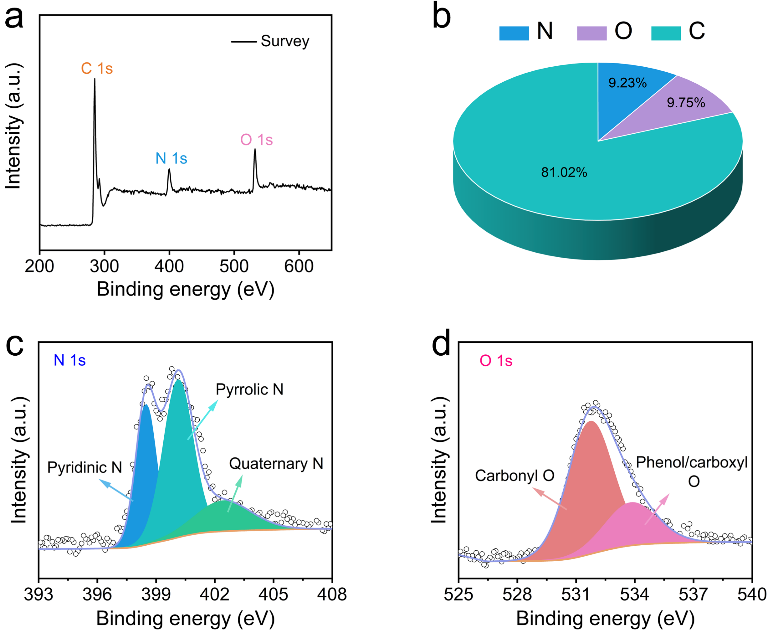


**Fig. S10 a** xps survey spectrum of PFC cathode. **b** Pie chart of element proportion. High-resolution **c** N 1s and **d** O 1s XPS spectra of PFC cathode.

**Notes to Fig. S10**: XPS spectrum identify the rich heteroatomic motifs of 9.23 at% N and 9.75 at% O for PFC (Fig. S10b).

**
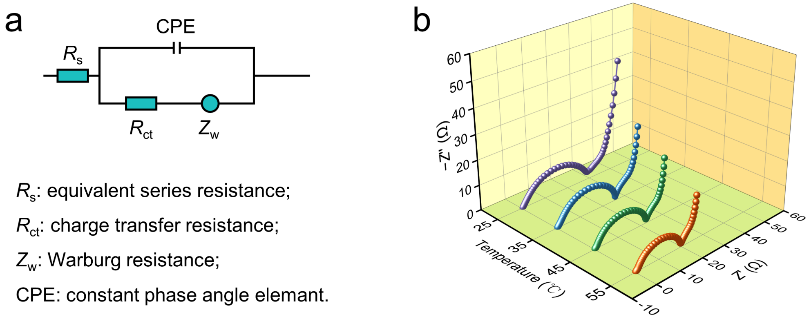
**

**Fig. S11 a** Equivalent circuit of Nyquist plots. **b** Electrochemical impedance spectroscopies (EIS) of the electrolyte at different operation temperatures.


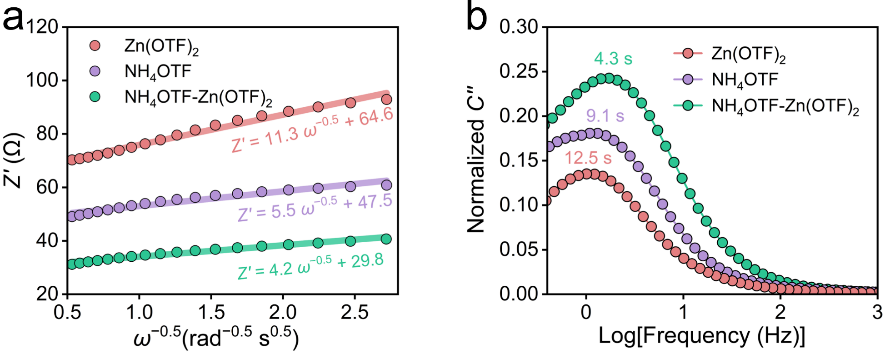


**Fig. S12 a** The relationship between *Z*' and *ω*^−0.5^ and **b** imaginary capacitance versus frequency of different electrolytes.

**Table S1** Comparison of electrochemical kinetics parameters of different electrolytes.

| Electrolyte | *E*_a1_ (kJ mol^−1^) | *E*_a2_ (kJ mol^−1^) | *R*_s_ (Ω) | *R*_ct_ (Ω) | *τ*_0_ (s) | σ (Ω s^−0.5^) |
| --- | --- | --- | --- | --- | --- | --- |
| Zn(OTF)_2_ | 9.9 | 26.4 | 2.5 | 70.1 | 12.5 | 11.3 |
| NH_4_OTF | 6.9 | 19.8 | 0.5 | 48.3 | 9.1 | 5.5 |
| Zn(OTF)_2_-NH_4_OTF | 4.3 | 11.5 | 0.3 | 27.8 | 4.3 | 4.2 |


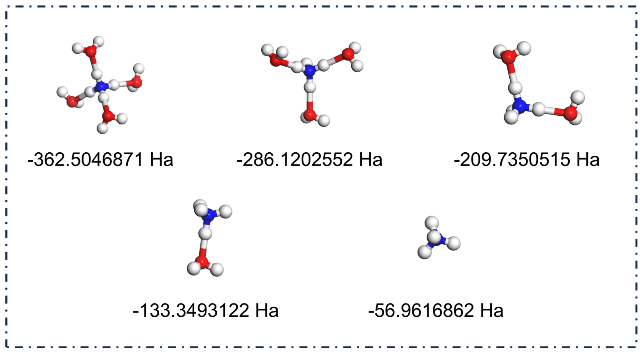


**Fig. S13** Optimized molecular structure models of hydrate NH_4_^+^ ion.


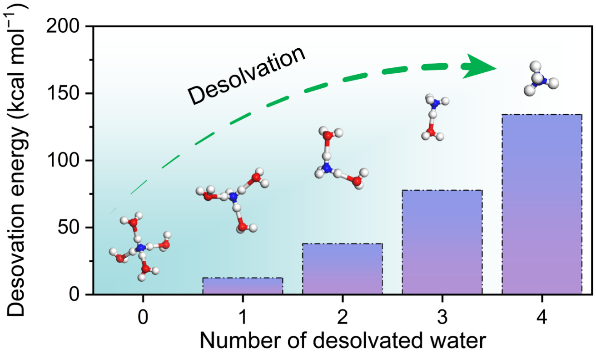


**Fig. S14** Schematic diagram of desolvation process and corresponding desolvation energy of NH_4_^+^ ion.


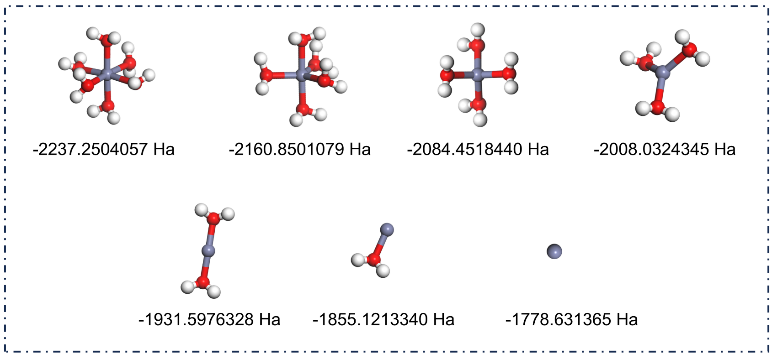


**Fig. S15** Optimized molecular structure models of hydrate Zn^2+^ ion.


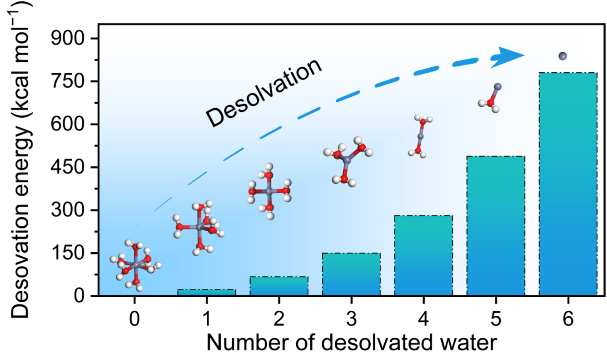


**Fig. S16** Schematic diagram of desolvation process and corresponding desolvation energy.


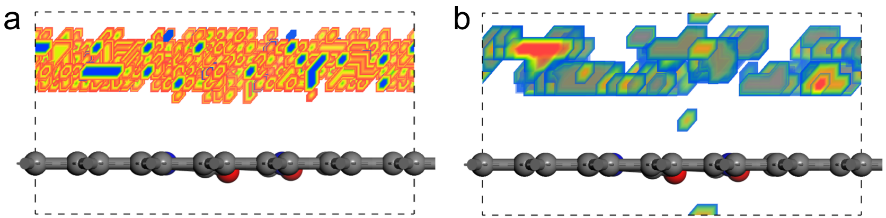


**Fig. S17** Side view of adsorption density isosurfaces: **a** Zn^2+^ and **b** NH_4_^+^.


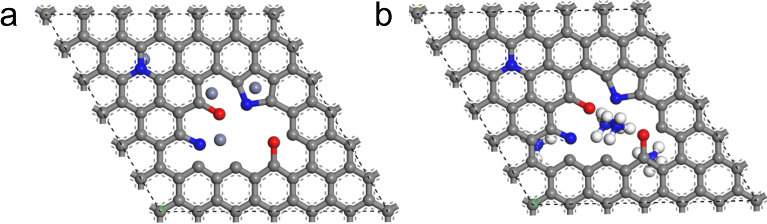


**Fig. S18** Snapshots of relative adsorption model of **a** Zn^2+^ and **b** NH_4_^+^.


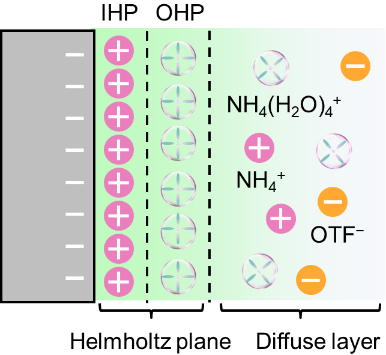


**Fig. S19** Modeling of interfacial adsorption in NH_4_OTF electrolyte.


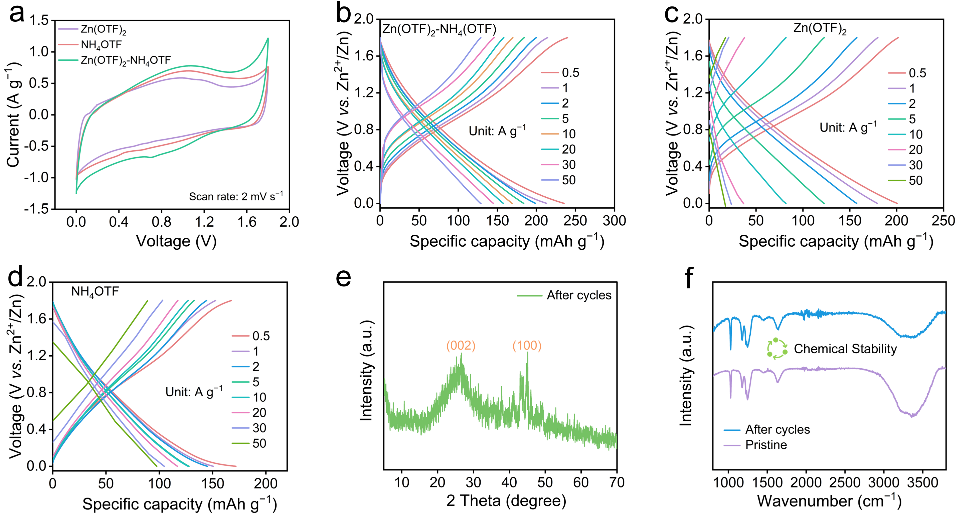


**Fig. S20** **a** CV curves of Zn||PFC capacitor in different electrolytes at 2 mV s^−1^. GCD profiles at various current densities of **b** Zn(OTF)_2_-NH_4_OTF, **c** Zn(OTF)_2_, and **d** NH_4_OTF. **e** XRD pattern of PFC cathode after cycles at Zn(OTF)_2_-NH_4_OTF electrolyte. **f** IR spectrum of Zn(OTF)_2_-NH_4_OTF electrolyte before and after cycles.

**Notes to Fig. S20**: IR spectra of Zn(OTF)_2_-NH_4_OTF hybrid electrolyte before and after cycles are identical, indicating its chemical stability and no side reactions during the electrochemical process. Furthermore, both PFC cathode (Fig. 4e and Fig. S20e) and Zn anode (Fig. S22) show excellent structural stability in Zn(OTF)_2_-NH_4_OTF hybrid electrolyte. Thus, the optimized Zn(OTF)_2_-NH_4_OTF hybrid electrolyte enables a stable electrochemical reaction environment for the desirable operation of high-performance Zn||PFC capacitor.


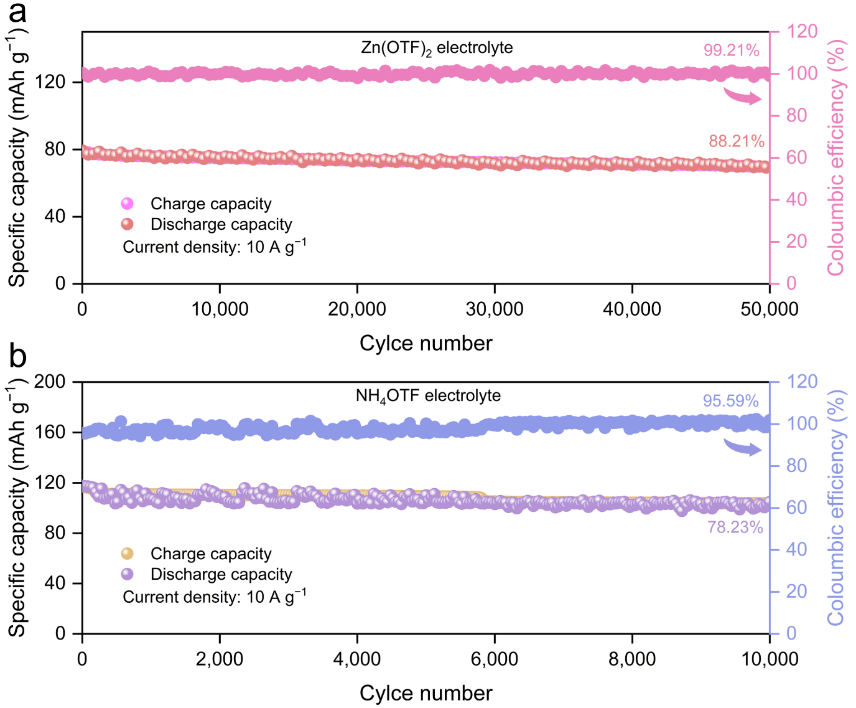


**Fig. S21** Cycling performance of Zn||PFC capacitor in **a** Zn(OTF)_2_ electrolyte and **b** NH_4_OTF electrolyte.

**
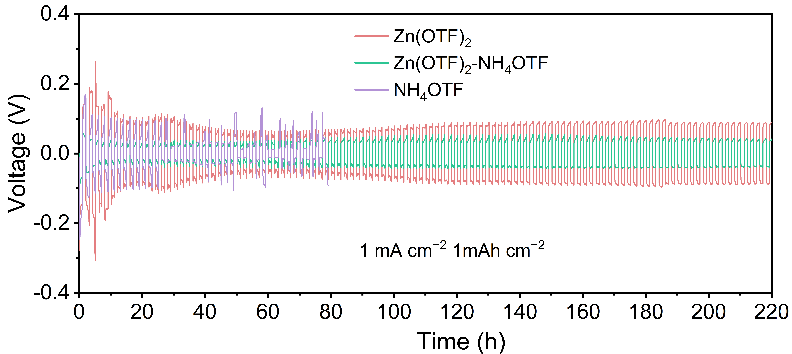
**

**Fig. S22** Long-term performance of Zn‖Zn cells at different electrolytes.

**Table S2** Comparison of rate capacity (*C*_m_), energy density (*E*), and cycling performance of recently reported carbon-based ZHCs in the literature.

| Cathode | Electrolyte | *C*_m_ (mAh g^–1^) | *E* (Wh kg^–1^) | Cycling lifespan | Refs. | |
| --- | --- | --- | --- | --- | --- | --- |
| PFC | 1 M Zn(OTF)_2_-  1 M NH_4_OTF | 237 @0.5 A g^–1^  130 @50 A g^–1^ | 147.2 | 98.6%, 400, 000 cycles,  30 A g^–1^ | This work | |
| PC | Gel/SA-acetate  electrolyte | 236.8@0.5 A g^–1^  87.3@50 A g^–1^ | 136.8 | 98.5%, 15, 000 cycles,  20 A g^–1^ | [S3] | |
| OCCs | Saturated  Zn(OTF)_2_ | 225@0.1 A g^–1^  71@20 A g^–1^ | 97.0 | 96.5%, 300, 000 cycles,  50 A g^–1^ | [S4] | |
| PC | 3 M Zn(ClO_4_)_2_ | 179.8@0.1 A g^–1^  78.4@20 A g^–1^ | 104.8 | 99.2%, 30, 000 cycles,  20 A g^–1^ | [S5] | |
| LVCR | 1 M Zn(OTF)_2_ | 166.7@0.2 A g^–1^  144.9@30 A g^–1^ | 126.6 | 96.9%, 20, 000 cycles,  10 A g^–1^ | [S6] | |
| SAC | 7.5 m ZnCl_2_ | 159.7@0.5 A g^–1^  72.2@20 A g^–1^ | 143.8 | 95.1%, 100, 000 cycles,  5 A g^–1^ | [S7] | |
| N-OPCNF | 1 M ZnSO_4_ | 136@0.1 A g^–1^  57@50A g^–1^ | 98.3 | 99.2%, 200, 000 cycles,  40 A g^–1^ | [S8] |  |
| PSC-  A600 | 1 M Zn(OTF)_2_ | 183.7@0.2 A g^–1^  81.8@20 A g^–1^ | 147.0 | 92.2%, 10, 000 cycles,  10 A g^–1^ | [S9] |  |
| AC | 2 M ZnSO_4_ | 132@0.2 A g^–1^  15@16 A g^–1^ | 140.8 | 72%, 20, 000 cycles,  4 A g^–1^ | [S10] |  |
| ACM-40 | polyacrylamide  ZnSO_4_ gel | 173@0.1 A g^–1^  41.7@20 A g^–1^ | 154.5 | 92.62%, 10, 000 cycles,  5 A g^–1^ | [S11] |  |
| MCHS | 2 M ZnSO_4_ | 174.4@0.1 A g^–1^  96.9@10 A g^–1^ | 129.3 | 96%, 10, 000 cycles,  1 A g^–1^ | [S12] |  |
| CS_30_ | 3 M Zn(OTF)_2_ | 265@0.5 A g^–1^  112@100 A g^–1^ | 161.2 | 96.8%, 400, 000 cycles,  10 A g^–1^ | [S13] |  |
| APC | 1 M ZnCl_2_ | 255.2@0.1 A g^–1^  93.1@10 A g^–1^ | 131.0 | 95.5%, 5, 000 cycles,  5 A g^–1^ | [S14] |  |
| AC | 60% EG  In 2 M ZnSO_4_ | 98@0.1 A g^–1^  61@20 A g^–1^ | 82.0 | 100%, 30, 000 cycles,  5 A g^–1^ | [S15] |  |


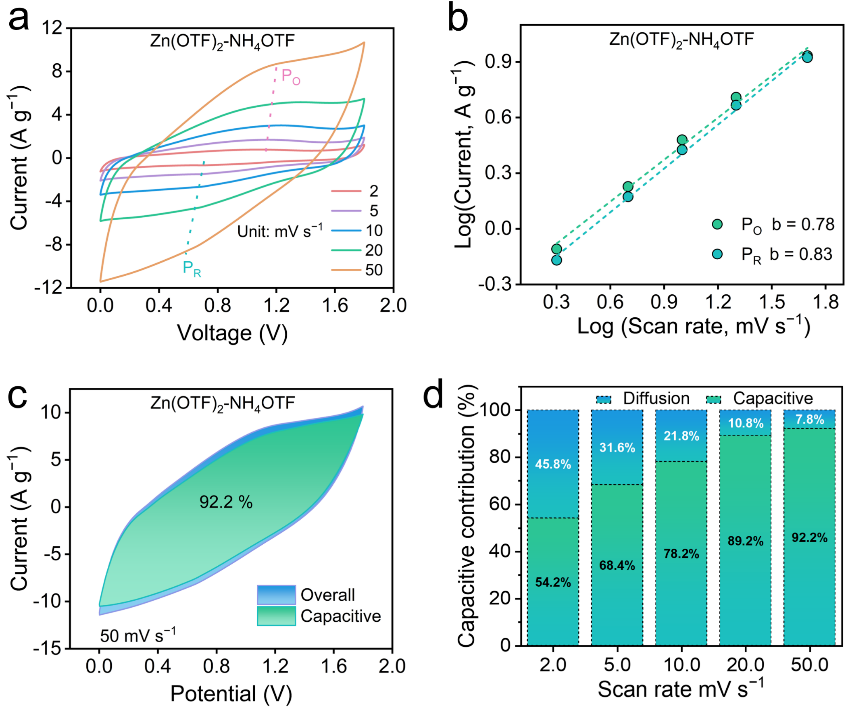


**Fig. S23** Investigation of charge storage kinetics in Zn(OTF)_2_-NH_4_OTF electrolyte. **a** CV profiles, **b** b values of P_O_ and P_R_, **c** capacitive contribution, and **d** normalized capacitive and diffusion-controlled contribution ratios at various scan rates.


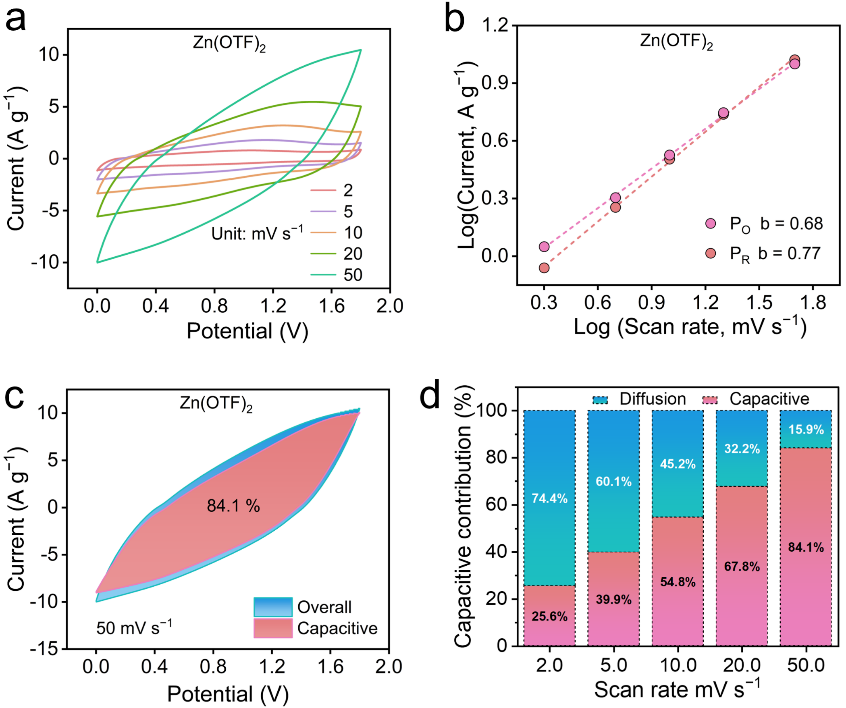


**Fig. S24** Investigation of charge storage kinetics in Zn(OTF)_2_ electrolyte. **a** CV profiles, **b** b values of P_O_ and P_R_, **c** capacitive contribution, and **d** normalized capacitive and diffusion-controlled contribution ratios at various scan rates.


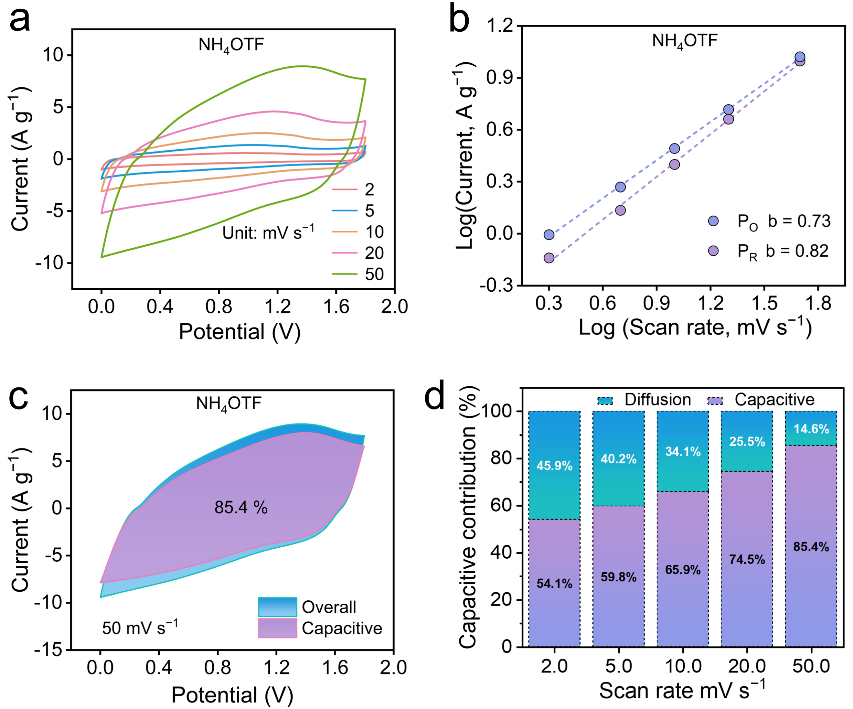


**Fig. S25** Investigation of charge storage kinetics in NH_4_OTF electrolyte. **a** CV profiles, **b** b values of P_O_ and P_R_, **c** capacitive contribution, and **d** normalized capacitive and diffusion-controlled contribution ratios at various scan rates.


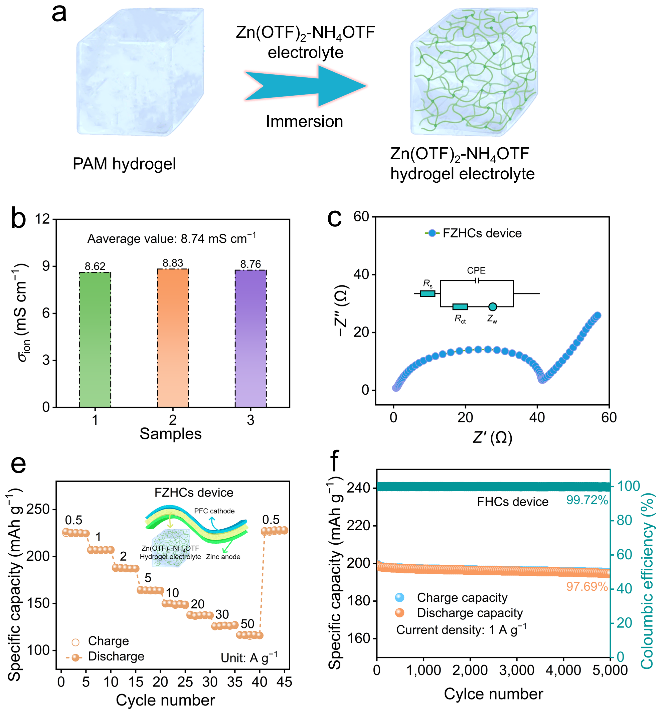


**Fig. S26 a** Schematic diagram of PAM hydrogel immersed in Zn(OTF)_2_-NH_4_OTF hybrid electrolyte. **b** Ionic conductivities of Zn(OTF)_2_-NH_4_OTF hydrogel electrolyte. **c** EIS spectrum and fitted Randles equivalent circuit of FZHCs. **e** Rate performance of FZHCs device. **f** Cycling stability of FZHCs device at 1A g^−1^.

**Notes to Fig. S26**: FZHCs show a low *R*_ct_ value of 40.2 Ω, implying fast interfacial ion diffusion kinetics (Fig. S26c). FZHCs exhibit high-rate capacities of 228.2 mAh g^−1^ at 0.5 A g^−1^ and 116.7 mAh g^−1^ at 50 A g^−1^ (Fig. S26e), together with a long cycling life of 5000 cycles with a capacity retention of 97.69% (Fig. S26f), highlighting the fast and stable interfacial electrode-electrolyte charge storage process.


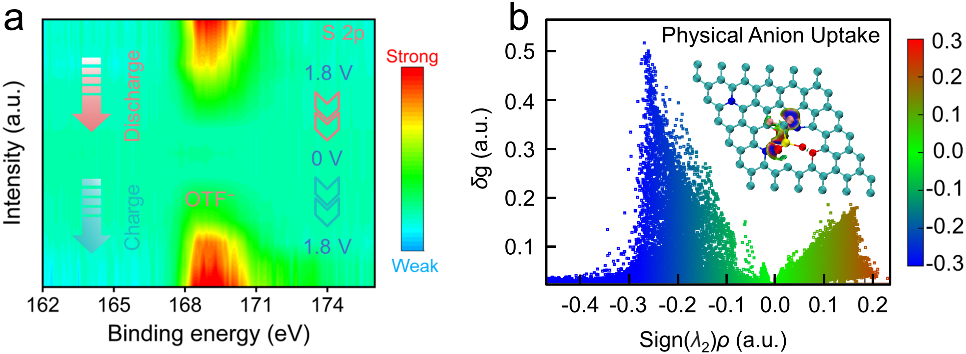


**Fig. S27** Experimental and theoretical simulations of OTF^−^ anion adsorpted in PFC cathode. **a** *Ex-situ* XPS spectra of OTF^−^ anion. **b** Plots of IGMH versus sign(*λ*_2_)*ρ* and corresponding gradient isosurfaces.


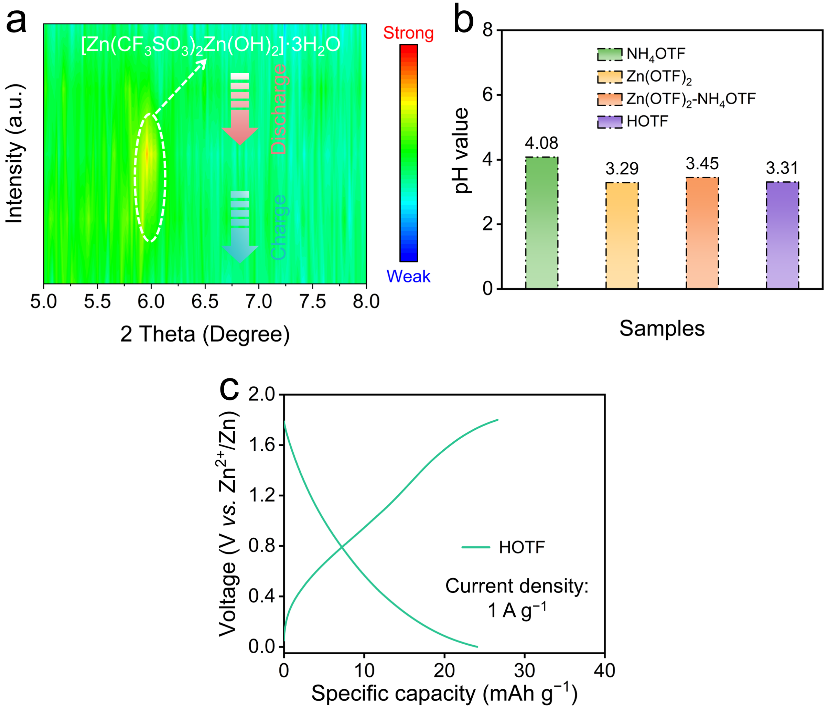


**Fig. S28 a** *Ex-situ* XRD patterns of Zn||PFC cell in Zn(OTF)_2_-NH_4_OTF electrolyte. **b** pH value of NH_4_OTF, Zn(OTF)_2_, Zn(OTF)_2_-NH_4_OTF and HOTF. **c** A GCD curve of Zn||PFC capacitor in HOTF electrolyte (pH=3.31).

**Notes to Fig. S28**: *Ex-situ* XRD pattern of PFC cathode shows the diffraction peak of [Zn(OTF)_2_Zn(OH)_6_]·3H_2_O (Fig. S28a), indicating that H^+^ takes part in the energy storage. PFC cathode in HCF_3_SO_3_/H_2_O electrolyte (pH=3.31, Fig. S28b) shows a capacity of 24.6 mAh g^−1^ in Zn||PFC capacitor (Fig. S28c), suggesting that H^+^ participates in the cathodic electrochemistry.


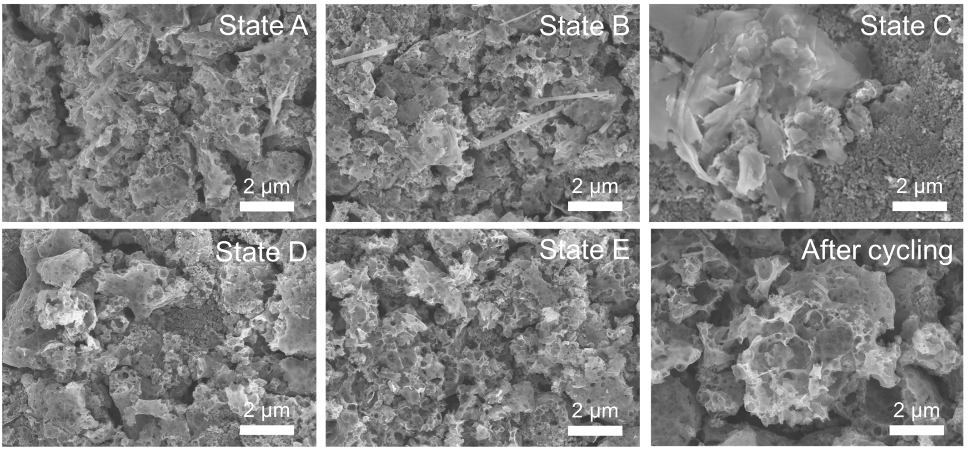


**Fig. S29** SEM images of PFC cathode at various (dis)charged states.

**
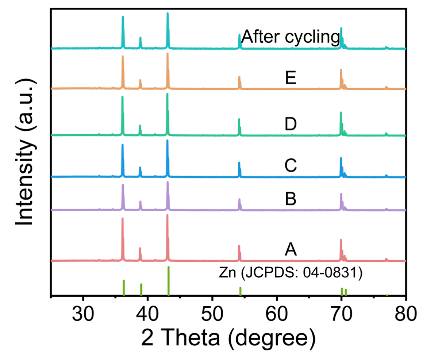
**

**Fig. S30** XRD patterns of the Zn anode at various (dis)charged states.


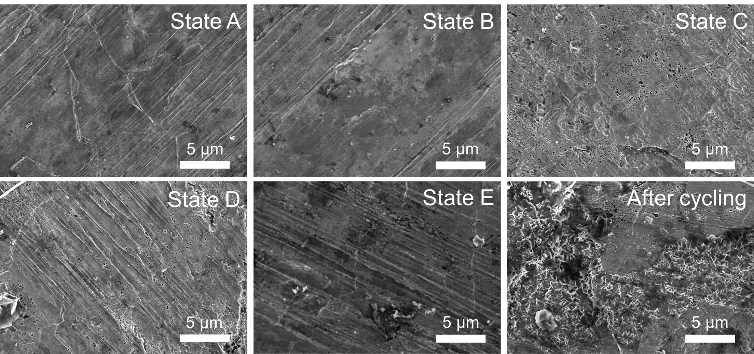


**Fig. S31** SEM images of the Zn anode at various (dis)charged states.


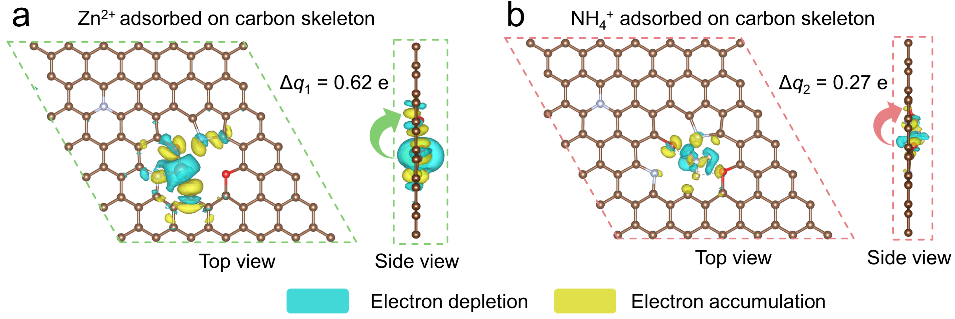


**Fig. S32** Differential electron density isosurface of single Zn^2+^ and NH_4_^+^ charge carrier adsorbed on the optimized carbon skeleton: **a** Zn^2+^ and **b** NH_4_^+^ ion.

**References**

[S1] H. Yao, Y. Li, Z. Chen, J. Chen, C.-F. Du, et al., Anion chemistry towards on-site construction of solid-electrolyte interface for highly stable metallic Zn anode. Angew. Chem. Int. Ed. **63**, e202411056 (2024). <https://doi.org/https://doi.org/10.1002/anie.202411056>

[S2] G. Zhang, L. Fu, Y. Chen, K. Fan, C. Zhang, et al., Hofmeister effects in supramolecular chemistry for anion-modulation to stabilize Zn anode. Adv. Mater. **36**, 2405949 (2024). <https://doi.org/https://doi.org/10.1002/adma.202405949>

[S3] C. Tian, J. Wang, R. Sun, T. Ali, H. Wang, et al., Improved interfacial ion migration and deposition through the chain-liquid synergistic effect by a carboxylated hydrogel electrolyte for stable zinc metal anodes. Angew. Chem. Int. Ed. **62**, e202310970 (2023). <https://doi.org/https://doi.org/10.1002/anie.202310970>

[S4] C.-C. Hou, Y. Wang, L. Zou, M. Wang, H. Liu, et al., A gas-steamed mof route to p-doped open carbon cages with enhanced Zn-ion energy storage capability and ultrastability. Adv. Mater. **33**, 2101698 (2021). <https://doi.org/https://doi.org/10.1002/adma.202101698>

[S5] J. Yin, W. Zhang, W. Wang, N. A. Alhebshi, N. Salah, et al., Electrochemical zinc ion capacitors enhanced by redox reactions of porous carbon cathodes. Adv. Energy Mater. **10**, 2001705 (2020). <https://doi.org/https://doi.org/10.1002/aenm.202001705>

[S6] K. Xiao, X. Jiang, S. Zeng, J. Chen, T. Hu, et al., Porous structure-electrochemical performance relationship of carbonaceous electrode-based zinc ion capacitors. Adv. Funct. Mater. **34**, 2405830 (2024). <https://doi.org/https://doi.org/10.1002/adfm.202405830>

[S7] C. Wang, Z. Pei, Q. Meng, C. Zhang, X. Sui, et al., Toward flexible zinc‐ion hybrid capacitors with superhigh energy density and ultralong cycling life: The pivotal role of ZnCl_2_ salt‐based electrolytes. Angew. Chem. Int. Ed. **60**, 990-997 (2020). <https://doi.org/10.1002/anie.202012030>

[S8] H. He, J. Lian, C. Chen, Q. Xiong, C. C. Li, et al., Enabling multi-chemisorption sites on carbon nanofibers cathodes by an in-situ exfoliation strategy for high-performance Zn-ion hybrid capacitors. Nano-Micro Lett. **14**, 106 (2022). <https://doi.org/10.1007/s40820-022-00839-z>

[S9] Z. Li, D. Chen, Y. An, C. Chen, L. Wu, et al., Flexible and anti-freezing quasi-solid-state zinc ion hybrid supercapacitors based on pencil shavings derived porous carbon. Energy Storage Mater. **28**, 307-314 (2020). <https://doi.org/https://doi.org/10.1016/j.ensm.2020.01.028>

[S10] Z. Wang, J. Huang, Z. Guo, X. Dong, Y. Liu, et al., A metal-organic framework host for highly reversible dendrite-free zinc metal anodes. Joule. **3**, 1289-1300 (2019). <https://doi.org/https://doi.org/10.1016/j.joule.2019.02.012>

[S11] Z. Wu, Y. Zuo, Y. Zhang, X. Li, J. Zhang, et al., Modulating inner helmholtz layer by electrocatalytically sieving [Zn(H_2_O)_6_]^2+^ for 10000-cycle zinc-ion hybrid capacitors under extremely harsh conditions. Energy Storage Mater. **70**, 103463 (2024). <https://doi.org/https://doi.org/10.1016/j.ensm.2024.103463>

[S12] P. Liu, W. Liu, Y. Huang, P. Li, J. Yan, et al., Mesoporous hollow carbon spheres boosted, integrated high performance aqueous Zn-ion energy storage. Energy Storage Mater. **25**, 858-865 (2020). <https://doi.org/https://doi.org/10.1016/j.ensm.2019.09.004>

[S13] Z. Song, L. Miao, L. Ruhlmann, Y. Lv, D. Zhu, et al., Lewis pair interaction self-assembly of carbon superstructures harvesting high-energy and ultralong-life zinc-ion storage. Adv. Funct. Mater. **32**, 2208049 (2022). <https://doi.org/https://doi.org/10.1002/adfm.202208049>

[S14] X. Shi, J. Xie, F. Yang, F. Wang, D. Zheng, et al., Compacting electric double layer enables carbon electrode with ultrahigh Zn ion storage capability. Angew. Chem. Int. Ed. **61**, e202214773 (2022). <https://doi.org/10.1002/anie.202214773>

[S15] N. Chang, T. Li, R. Li, S. Wang, Y. Yin, et al., An aqueous hybrid electrolyte for low-temperature zinc-based energy storage devices. Energy Environ. Sci. **13**, 3527-3535 (2020). <https://doi.org/10.1039/D0EE01538E>
